# Occurrence of Phenotypic Multidrug-Resistant *E. coli* in Kentucky (USA) Surface Waters and Exploration of Sentinel Antibiotics for One Health Surveillance

**DOI:** 10.3390/antibiotics15070709

**Published:** 2026-07-21

**Authors:** Jason W. Marion, S. Travis Altheide

**Affiliations:** 1 Environmental Health Science & Sustainability Program, College of Health Sciences, Eastern Kentucky University, Richmond, KY 40475, USA; 2 Medical Laboratory Science Program, College of Health Sciences, Eastern Kentucky University, Richmond, KY 40475, USA

**Keywords:** antibiotic resistance, multidrug resistance, *E. coli*, fecal indicator bacteria, one health, *Enterobacter*, environmental surveillance

## Abstract

**Background/Objectives**: Antimicrobial resistance (AMR) is a global public health threat, and surface waters may serve as surveillance locations for multidrug-resistant (MDR) bacteria within communities. This study explored MDR *E. coli* in Kentucky (USA) surface waters, primarily evaluating non-susceptibility to tetracycline, for assessing MDR associations. **Methods**: Surface water samples were collected from 47 sites in East-Central Kentucky during 2022 and 2024. Isolates were obtained using selective differential media, with and without tetracycline (TE) supplementation, targeting *E. coli* and TE-resistant *E. coli.* Species identification and antimicrobial susceptibility testing against 21 antibiotics representing 13 categories was performed with recovered presumptive *E. coli* isolates. MDR was defined as non-susceptibility to at least one antibiotic in three or more antimicrobial categories. **Results**: Of 151 isolates, 130 (86%) were identified as *E. coli*. Overall, 25% and 13% of recovered *E. coli* isolates from media with and without TE had MDR phenotypes, respectively, which included one isolate with non-susceptibility to 10 of 12 designated antibiotic categories. MDR prevalence was higher among incidentally recovered *Enterobacter* spp. (100%) than *E. coli*. TE non-susceptibility occurred in 100% and 18% of *E. coli* isolates recovered from TE-treated and untreated media, respectively. Ampicillin non-susceptibility occurred in 28% and 16% of *E. coli* isolates from TE-treated and untreated media. Among this non-random set of recovered isolates, ampicillin and cefazolin non-susceptibility demonstrated potential for MDR *E. coli* prediction. TE-treated media did not significantly improve MDR *E. coli* recovery. **Conclusions**: MDR *E. coli* and one potentially extensively drug-resistant (XDR) *E. coli* isolate were recoverable from Kentucky surface waters. Ampicillin and cefazolin non-susceptibility demonstrated potential as sentinel markers for informing screening media development for AMR surveillance in Kentucky waters.

## 1. Introduction

Antimicrobial resistance (AMR) is a persistent global public health threat. In 2021, bacterial AMR was associated with an estimated 4.71 million deaths worldwide, including approximately 1.14 million deaths directly attributable to resistance, whereby the excess mortality would not have occurred if drug-resistant infections had instead been drug susceptible [1]. Although the overall burden is lower in the Americas, approximately 200,000 deaths annually are associated with AMR, with 50,000 deaths directly attributable to AMR infections [2]. Enteric bacteria commonly detected in fecal-contaminated environments account for much of this burden in the Americas, including *Escherichia coli* (109,000 associated deaths), *Klebsiella pneumoniae* (66,100), *Enterobacter* spp. (18,500), *Serratia* spp. (3980), and *Citrobacter* spp. (2330) [2]. Collectively, these organisms highlight Enterobacterales as major contributors to AMR-associated morbidity and mortality.

In the United States, the Centers for Disease Control and Prevention (CDC) reported increasing hospital-associated infections linked to enteric bacteria in 2019, particularly extended-spectrum β-lactamase (ESBL)–producing Enterobacterales and vancomycin-resistant *Enterococcus* (VRE) [3]. Updated CDC surveillance for 2021–2022 indicates these infections have continued to increase [4]. Consequently, AMR prevention strategies are increasingly adding focus to identifying environmental reservoirs of resistant bacteria and the conditions that support AMR emergence, persistence, and transmission. Aquatic environments influenced by human activities, where domestic, industrial, hospital, agricultural, wildlife, and animal wastes co-mingle, have been implicated as key settings for AMR maintenance and spread [5].

Surface water monitoring is therefore recognized as an important component of AMR surveillance, particularly within a One Health framework. Watersheds integrate diverse inputs such as wastewater effluent, stormwater runoff, agricultural discharges, wildlife fecal inputs, and soil erosion, making surface waters effective sentinels of community-level AMR pressures. Reflecting this, the National Antimicrobial Resistance Monitoring System (NARMS) Strategic Plan (2021–2025), jointly authored by the US Department of Agriculture (USDA), Food and Drug Administration (FDA), and CDC, called for geographically representative surface water monitoring to establish baseline AMR data [6]. To support this goal, the NARMS Environmental Working Group (EWG), coordinated by the US Environmental Protection Agency (EPA), identified key research priorities, including (1) establishing baseline AMR in U.S. freshwater systems, (2) enabling spatial and temporal comparisons, (3) quantifying AMR bacteria using colony forming unit (CFU) or most probable number (MPN) approaches to support risk assessment, and (4) characterizing resistance gradients across land use types [7].

Effective environmental AMR surveillance requires indicator organisms that are ubiquitous, feasible to measure, sensitive to change, and interpretable for risk assessment. The NARMS EWG includes in their recommendations culture-based approaches using EPA-approved methods such as EPA Method 1603 [8] and the Idexx Colilert system [9] for enumerating *E. coli* in surface waters [7]. These methods enable isolate recovery and direct phenotypic assessment of resistance to antibiotics and antibiotic categories of interest. Although molecular methods provide valuable insights into resistance genes, phenotypic resistance remains the standard for clinical and regulatory decision-making by the FDA, CLSI, and EUCAST [10,11].

*E. coli* has long been used as an indicator of fecal contamination and enteric disease risk in drinking [12], irrigation [13], and recreational waters [14,15]. Recent evidence further suggests that *E. coli* may serve as a proxy for monitoring AMR and the gut resistome due to similar antimicrobial selection pressures in human and animal populations [16]. A study comparing tetracycline (TE) resistance in *E. coli* isolates from human stool and adjacent rivers found no significant differences in resistance prevalence [17]. Enumerating both total and resistant *E. coli* populations has therefore been proposed to normalize resistance counts and assess the role of fecal loading in AMR emergence and transmission [7,11]. Several studies have used TE-containing media to enumerate TE-resistant *E. coli* using a most probable number (MPN) method for estimating the proportion of culturable *E. coli* that are TE-resistant [18,19,20,21]. For example, 100 mL of sample is mixed with TE-treated media and 100 mL is mixed with untreated media. Then, by comparing the two obtained MPN values, often per 100 mL, estimation of the proportion of culturable *E. coli* that is TE-resistant is possible.

TE resistance has been presumed suitable for environmental AMR surveillance because tetracyclines are among the most widely used antibiotics globally, particularly in food-animal production [22,23]. Global point-prevalence surveys indicate TE resistance is the most common phenotype in food-animal isolates, with a mean prevalence of 59% across countries [24]. Tetracyclines are relatively stable in aquatic systems [22], which may contribute to the widespread persistence of TE-resistant *E. coli* [25]. In U.S. surface waters, TE resistance prevalence has ranged from 8.8% in the agriculturally dominated Maumee River (Ohio) to 27% in Texas runoff from pasture, prairie, and cropland [26,27]. Irrigation water studies in Maryland using Colilert media with and without tetracycline reported resistant *E. coli* MPN levels of up to 12.7% and 6.3% at TE concentrations of 4 μg/mL and 16 μg/mL, respectively [21].

TE resistance has been proposed as an indicator of broader antibiotic resistance [28], while co-resistance to TE and amoxicillin (AMO) has been identified as an efficient marker of multidrug resistance (MDR) in *E. coli* [29]. Similarly, aquaculture studies have reported co-resistance to AMO, TE, and either ciprofloxacin (CP) or sulfamethoxazole–trimethoprim (S/T) as indicative of MDR [30].

Given the limited data on phenotypic AMR from Kentucky surface waters, this study aimed to determine whether TE-treated media enhances recovery of MDR isolates compared with non-selective media. Because Kentucky consistently ranks among the top three U.S. states for oral antibiotic prescribing (1089 prescriptions per 1000 persons in 2024) [31], we additionally sought to characterize AMR and MDR prevalence among the recovered *E. coli* isolates from small streams located in Central and East-Central Kentucky.

## 2. Results

### 2.1. Origin of Isolates and Associated Microbiological-Based Water Quality

A total of 151 magenta-colored isolates were recovered from m-TEC ChromoSelect agar plates for antibiotic susceptibility testing in 2022 and 2024. Recovered isolates from m-TEC originated from isolation streaks using broth suggestive of *E. coli* growth from individual wells of 96-well plates or quanti-trays, which were used as part of the ColiGlow [19] or Idexx Colilert [9] MPN methods. Overall, 70 (46%) of the recovered isolates obtained were from ColiGlow, 58 (38%) from ColiGlow containing TE, and 23 (15%) from Colilert media. Prior to antibiotic susceptibility testing, all 151 isolates were incubated in 1 mL of Colilert broth and evaluated to confirm presumptive *E. coli* identification.

The recovered isolates were from 12 sites in 2022 and 39 sites in 2024 (Table 1). Given that some 2022 sites were re-used in 2024, the total number of unique sampling sites was 47. While a convenience sampling approach was used, isolate analysis for this study on antibiotic susceptibility was biased towards samples in which presumed non-susceptibility to TE was observed (from ColiGlow containing TE) for the purpose of evaluating the TE-treated ColiGlow in selecting for TE non-susceptible isolates and MDR isolates.

Among the 12 sampling sites in 2022, when combining repeated visits, the number of isolates obtained per site ranged from 1 to 5 (mean = 2.50, median = 2). Most sites in 2022 were visited twice, with one isolate being recovered from one positive well of the 96-well plates for ColiGlow and ColiGlow + TE. In 2024, the number of isolates studied per site ranged from 1 to 12 (mean = 3.26, median = 2.5), with 12 being from the university farm site that was sampled weekly in 2024. Most sites in 2024 were visited once. Additionally, in 2024, 23 isolates were from individual wells from 23 different quanti-trays from 18 different sites (Table 1).

In addition to the university’s farm site, several sites were sampled twice, typically several weeks later, to introduce greater variability. For optimizing land use variability, samples were collected from predominantly first- and second-order headwater streams, with several third-order tributaries, across the Upper Kentucky River and Licking River watersheds within the Ohio River Basin. Most water samples, and their subsequent isolates, were obtained from within Madison County (Table 1) as well as Estill and Rowan Counties in Kentucky, where the land cover varies considerably between pastureland, deciduous forest, developed land, and a mixture of these land covers (Figures S1–S3).

In evaluating the microbiological-based water quality associated with all isolates recovered in the study (*n* = 151), the median *E. coli* density was 959 MPN per 100 mL (Table 2). In total, 62 (41%) of all 151 isolates studied were from water samples that exceeded the method range of 1479 MPN per 100 mL; all 96 wells for the media without TE were positive for presumptive *E. coli*. Among the 130 isolates identified as *E. coli*, 87 (67%) and 109 (72%) were obtained from water samples which had *E. coli* densities exceeding single-day (235 MPN per 100 mL) and 30-day geometric mean (130 MPN per 100 mL) microbiological-based water quality guidelines for primary contact recreation as set by the U.S. EPA and Commonwealth of Kentucky [14,32]. Among the other (non-*E. coli*) isolates studied, 15 (71%) of 21 exceeded both the 130 and 235 MPN per 100 mL guidelines.

When stratified by media with and without TE, the *E. coli* isolates recovered from untreated media (*n* = 77) were more frequently obtained from water samples with *E. coli* levels within range (<1479 MPN per 100 mL) compared to isolates recovered from TE-treated media (*n* = 53). The proportion of samples in which all wells in the 96-well plate were positive for presumptive *E. coli* (overrange) was significantly higher (*p* < 0.001) for isolates originating from TE-treated media (57%) versus untreated media (15%). All isolates were presumptive *E. coli* when obtained from the selective differential media used and after subsequent examination in Colilert broth. However, upon performing species identification, there was a significant difference in confirmed *E. coli* identification between samples exceeding or not exceeding the method range (>1479 MPN per 100 mL). Among the 130 isolates confirmed as *E. coli*, 49 (38%) originated from samples exceeding the method range. In contrast, 13 (62%) of the 21 isolates identified as non-*E. coli* came from samples exceeding the method range. This difference was statistically significant (*p* = 0.036), indicating that when fecal indicator bacteria concentrations exceeded the method range, isolates recovered from the selective differential media had a greater likelihood of being a non-target species.

The 53 isolates identified as *E. coli* from TE-treated media were obtained from the positive wells of the ColiGlow + TE method, which enumerates TE-resistant *E. coli.* It was apparent that these isolates were from samples in which TE treatment effectively selected for TE-resistant and TE-non-susceptible *E. coli*, as evident by the considerable reduction or elimination of all susceptible *E. coli*, as most, if not all, of the 96 wells had no evidence of growth (resistant and non-susceptible are defined in Section 4). Specifically, in the TE-treated media, the median *E. coli* density of 77.5 MPN per 100 mL was noticeably lower than the median (>1479 MPN per 100 mL) of the paired ColiGlow method results, where no TE was added to media (Table 2). In a paired analysis focusing on the microbiological water quality associated with the 58 samples from which TE-treated isolates were recovered, the median and mean densities of presumed TE-non-susceptible *E. coli* were 77.5 and 261 MPN per 100 mL, respectively, whereas the mean and median density of all presumed *E. coli* in the same water sample was >1479 MPN per 100 mL for both. Among all 58 samples, 54 of the paired comparisons presented higher total *E. coli* density in MPN per 100 mL than the presumed TE-non-susceptible *E. coli* density in the water. There were also two ties and two with higher presumed TE-non-susceptible levels. Accordingly, there was a significant difference in the median total and TE-non-susceptible presumed *E. coli* densities, demonstrating a treatment effect in reducing total *E. coli* (*p* < 0.0001).

### 2.2. Antibiotic Susceptibility Profile

**Figure 1:**
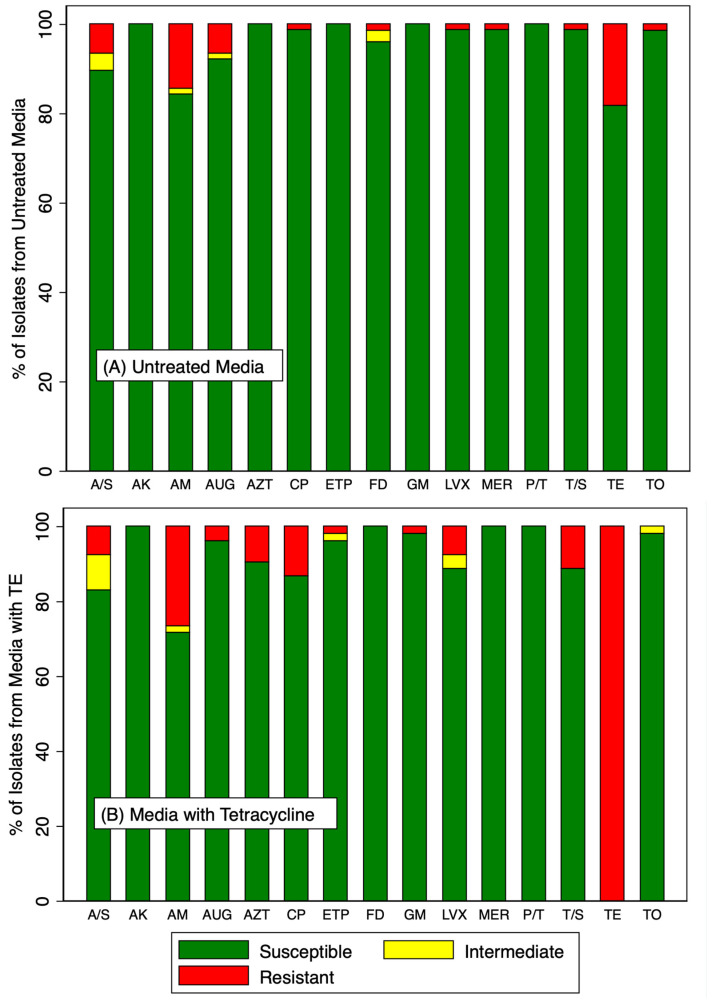
Antibiotic susceptibility profiles presented as stacked bar charts illustrating the percentage of *E. coli* isolates exhibiting susceptibility, intermediate resistance, and resistance to 15 antibiotics. (**A**) Antibiotic susceptibility profile for *E. coli* isolates obtained from media without tetracycline (*n* = 77); (**B**) antibiotic susceptibility profile for *E. coli* isolates obtained from tetracycline-treated media (*n* = 52). A/S: ampicillin–sulbactam, AK: amikacin, AM: ampicillin, AUG: amoxicillin–clavulanic acid, AZT: aztreonam, CP: ciprofloxacin, ETP: ertapenem, FD: nitrofurantoin, GM: gentamicin, LVX: levofloxacin, MER: meropenem, P/T: piperacillin–tazobactam, T/S: trimethoprim–sulfamethoxazole, TE: tetracycline, TO: tobramycin.

**Figure 2:**
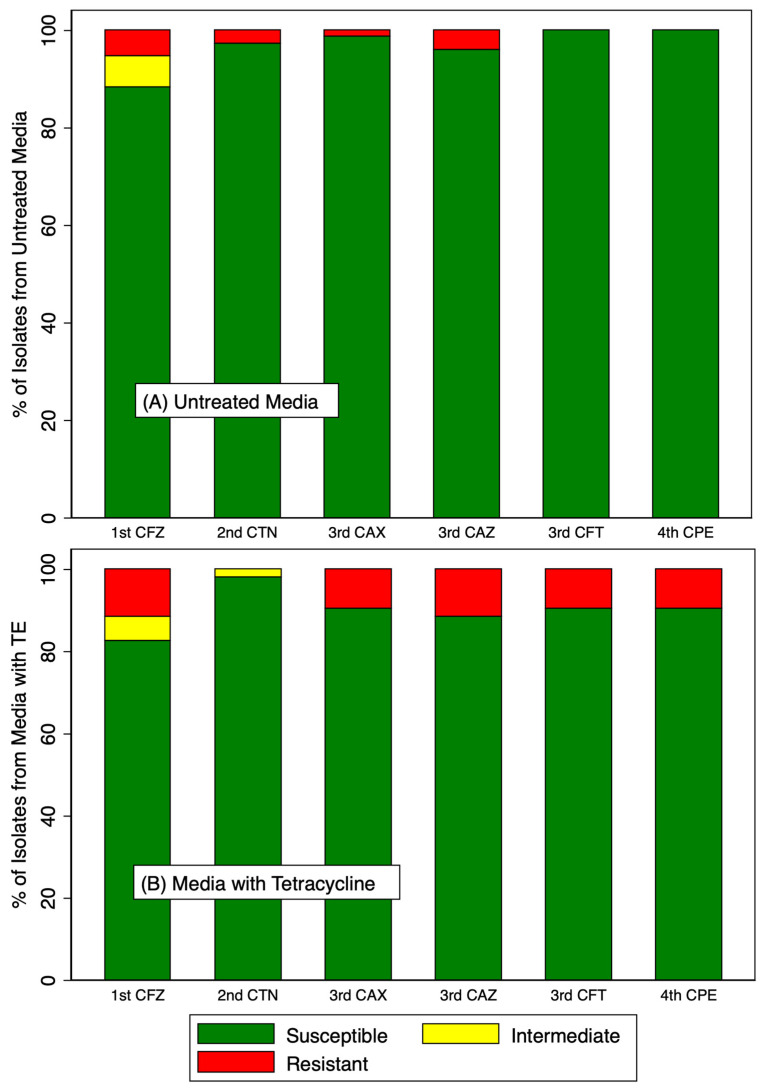
Cephalosporin-specific antibiotic susceptibility profiles for *E. coli* isolates evaluated, illustrating the percentage of isolates exhibiting susceptibility, intermediate resistance, and resistance to six antibiotics. (**A**) Cephalosporin susceptibility profile for isolates obtained from media without tetracycline (*n* = 77); (**B**) cephalosporin susceptibility profile for isolates obtained from tetracycline-treated media (*n* = 52). CFZ: cefazolin, CTN: cefotetan, CAX: ceftriaxone, CAZ: ceftazidime, CFT: cefotaxime, CPE: cefepime.

Antibiotic susceptibility profiles for isolates from the untreated and TE-treated media show several antibiotic-specific similarities and differences (Figure 1 and Figure 2). Non-susceptibility to TE was very common, occurring in 66 (52%) of the 128 *E. coli* isolates (Table S1) and 73 (48%) of all 151 isolates, which includes the non-*E. coli* isolates that presented like *E. coli* in Colilert (Table S2). As expected, TE non-susceptibility occurred in all 53 (100%) *E. coli* isolates recovered from media containing TE (Figure 1), which was significantly greater (Holm adjusted *p* < 0.019) than the 18% prevalence (14 of 63) of TE non-susceptibility among *E. coli* isolates from the media without TE (Table S3).

Other than for TE, no significant differences (*p_adj_* > 0.05) were observed for the prevalence of non-susceptibility to the other antibiotics for *E. coli* isolates obtained from TE-treated and untreated media upon accounting for the multiple comparisons. In singular comparisons, unadjusted Fisher’s Exact Test *p*-values demonstrated significantly (*p* < 0.05) higher prevalences of non-susceptibility among *E. coli* from TE-treated media versus untreated media to ceftriaxone, cefotaxime, cefepime, aztreonam, ciprofloxacin, levofloxacin, and trimethoprim-sulfamethoxazole. However, upon employing a Holm–Bonferroni correction to the *p*-values accounting for multiple testing, there was insufficient evidence (and/or statistical power) to observe significant differences in non-susceptibility prevalences between *E. coli* isolates from TE-treated and untreated media to all the antibiotics assessed other than TE (Holm adjusted *p* > 0.05; Table S3).

Without the TE selection pressure in the untreated media, TE non-susceptibility remained the most prevalent (18%) among *E. coli* isolates, followed by ampicillin (AM) non-susceptibility (16%) (Figure 1; Table S2). Interestingly, when including the non-*E. coli* isolates, the prevalence of TE non-susceptibility increased to 25% (Table S4), demonstrating a higher level of TE non-susceptibility among the breakthrough organisms than *E. coli*.

Between the two treatment groups (with and without TE), the prevalence of AM non-susceptibility among untreated media isolates (16%) was not significantly different from the 28% prevalence of AM non-susceptibility among isolates from the TE-treated media (adjusted *p* = 0.869; Table S2). Like AM, ampicillin–sulbactam (A/S) non-susceptibility was also relatively more common (10%) compared to the frequency of non-susceptibility among all other antibiotics assessed for *E. coli* isolates. Only TE, AM, A/S and cefazolin (CFZ) exceeded 10% non-susceptibility among *E. coli* isolates from untreated media. When incorporating all the isolates studied, including the non-*E. coli* organisms, the non-susceptibility prevalence >10% among untreated media isolates then included amoxicillin–clavulanic acid (AUG), nitrofurantoin (FD), and cefotetan (CTN) (Table S4).

Among cephalosporins, non-susceptibility to the first-generation cefazolin (CFZ) was relatively common for *E. coli* isolates, with 17% and 12% prevalence between the TE-treated and untreated groups (Figure 2). Non-susceptibility to cefotetan (CTN) was less than 3% among the *E. coli* isolates, but when focusing on all isolates, it exceeded 10% among the untreated media (Table S4).

Several antibiotics were effective against all or nearly all isolates evaluated. Of all isolates evaluated, all were susceptible to amikacin (AK) and piperacillin–tazobactam (P/T) (Tables S1 and S2). Over 99% of all isolates were susceptible to gentamicin (GM) and meropenem (MER). Over 98% of isolates were susceptible to tobramycin (TO) and ertapenem (ETP).

### 2.3. Species Composition of Isolates Obtained

All isolates evaluated were presumptive *E. coli* based upon four factors after 24 h of incubation: (1) fluorescence under longwave UV in ColiGlow broth; (2) colony appearance as magenta, dark purple, or maroon on mTEC agar; (3) their ability to generate yellow coloration in Idexx Colilert broth under natural light; and (4) their ability to produce fluorescence in Colilert broth under longwave UV light after re-inoculation from agar plates. Among the 151 isolates evaluated in this study, the 30 presumptive *E. coli* isolates analyzed in 2022 were identified only by their biochemical profile in tandem with their antibiotic susceptibilities as conducted using the Microscan instrument. The remaining 121 isolates studied in 2024 were identified by both the Microscan and MALDI-TOF-MS methods.

All isolates were identified to the genus as members of the order Enterobacterales, with the isolation approach directed towards the selection of *E. coli* (85% of recovered isolates). In evaluating the two treatment groups from which the isolates originated, 52 (90%) of 58 isolates from TE-treated media were *E. coli* vs. 77 (83%) of 93 isolates from media without TE, which is not significantly different (*p* = 0.245). When further stratifying the untreated media for isolate origination, it was observed that 2 (9%) of the 23 isolates from Colilert were *Klebsiella* spp., and 14 (20%) of 70 ColiGlow isolates were not *E. coli*. There was insufficient evidence to observe a significant difference (*p* = 0.341) in the two methods related to correct *E. coli* classification.

For several genera presenting as *E. coli* in the origination media used, notably *Citrobacter*, *Enterobacter*, *Klebsiella*, *Kluyvera*, *Morganella*, and *Salmonella*, there was some uncertainty and/or disagreement between the MicroScan and MALDI-TOF-MS (MALDI) methods for identifying *E. coli*. Among the 121 MicroScan results that were paired with MALDI identification, eleven (9%) had discordant species identification for *E. coli* (Table 3). Interestingly, MicroScan biochemically identified *Morganella morganii* (99.9% probability), *Kluyvera ascorbata* (83.2% probability), and *Salmonella enterica* (75% probability); MALDI identified each of these isolates as *E. coli* and they were therefore analyzed in the data as *E. coli*. Among 108 isolates identified as *E. coli* by MALDI, the MicroScan agreed with 99 (92%). Similarly, among the 102 isolates identified by the MicroScan as *E. coli*, MALDI agreed with 99 (97%).

Among the 30 isolates relying on identification only by MicroScan before 2024, 20 (67%) were *E. coli*, six (20%) were *Enterobacter cloacae*, three (10%) were *Klebsiella* spp., and one was *Serratia odorifera* (3%). Using the 2024 isolates to assess agreement between MicroScan and MALDI with *E. cloacae*, three (75%) of the four MicroScan isolates were identified as *Enterobacter* spp., and one (25%) was identified by MALDI as *E. coli.* Among *Enterobacter* spp., MALDI had difficulty in discerning members of the *Enterobacter cloacae* complex (ECC), specifically *E. hormaechei*, *E. cloacae*, and *E. asburiae.* At the genus level, one (25%) of four isolates identified as *E. cloacae* by Microscan was identified as *E. cloacae* by MALDI. For the other two, MALDI could not differentiate between ECC members.

### 2.4. Prevalence of Multidrug Resistance Among Recovered Isolates

#### 2.4.1. Differences in Multridrug Resistance Among *E. coli* and Non-*E. coli* Isolates

Upon evaluating susceptibility for thirteen antibiotic categories, 18% of *E. coli* isolates presented as MDR (non-susceptibility to ≥3 categories) (Table 4). The spatial distribution of all MDR isolates are viewable on county-level (Figures S3–S5) and state-level maps (Figure S6), illustrating that MDR isolates were dispersed across multiple sampling areas and are represented mostly in Madison County, which was also the most intensely sampled county. The 100% prevalence of MDR among recovered isolates of *Enterobacter* spp. was significantly greater (*p* < 0.001) than among recovered *E. coli* isolates.

In assessing whether the media treated with TE was associated with a higher recovery of MDR *E. coli* isolates, no difference was observed. Specifically, the prevalence of MDR among recovered *E. coli* isolates from untreated media was 13%, whereas the prevalence was 25% among *E. coli* isolates from the TE-treated media (Tables S5 and S6). While the *E. coli* isolates from TE-treated media had a higher MDR prevalence than the untreated media, the difference in MDR prevalence was not significantly different (*p* = 0.104). In evaluating the impact of TE-treatment on the recovery in *Enterobacter* isolates, which had high MDR prevalence, *Enterobacter* isolates were observed as seven (7.7%) of the 91 isolates from media without TE and in two (3.4%) of 58 isolates from TE-treated media. The prevalence of recovery of *Enterobacter* was not significantly different (*p* = 0.483) between media types.

#### 2.4.2. Prevalence and Description of Extensively Drug-Resistant *E. coli*

Among all MDR isolates, one *E. coli* isolate was potentially extensively drug-resistant (XDR), defined herein as non-susceptibility to all but two of the 12 categories of antimicrobials for *Enterobacteriaceae* (Table 4). This isolate was recovered from the media containing TE. The isolate was susceptible to all three aminoglycosides (gentamicin, tobramycin, and amikacin) and the one antipseudomonal penicillin + β-lactamase combination (piperacillin-tazobactam). Additionally, the isolate was susceptible to nitrofurantoin, which is excluded from the list of antibiotic categories for determining MDR and XDR as specified in Magiorakos et al. [33]. While susceptible to meropenem, the isolate was not susceptible to ertapenem. The MicroScan Panel Alert Report for this isolate, inclusive of the biochemical results and minimum inhibitory concentration (MIC) output, is provided as Supplementary File S1.

### 2.5. Evaluation of Microbiological-Based Water Quality Impact on Multidrug Resistance

The prevalence of MDR among the recovered isolates relative to three levels of microbiological water quality (as determined by *E. coli* densities) is presented in Table 5. Using categories defined as EC-1 (<100 *E. coli* MPN per 100 mL), EC-2 (100 to <1479 MPN per 100 mL), and EC-3 (≥1479 MPN per 100 mL), there is insufficient evidence in our data to suggest the prevalence of MDR among *E. coli* isolates was linked to the microbiological water quality of the sample from which they were obtained. While not significant, the prevalence of MDR among *E. coli* isolates was higher among isolates recovered from the water samples with <100 *E. coli* MPN per 100 mL than the samples exceeding the method range (Table 5). Similar comparisons examining the prevalence of non-susceptibility when water samples exceeded the methodological range are available for *E. coli* isolates and all isolates in Tables S7 and S8, wherein no significant differences are observed.

### 2.6. Diagnostic Performance of Non-Susceptibility to Individual Antibiotics for Predicting MDR

The diagnostic performance of the binary outcome (non-susceptibility) for each antibiotic was evaluated for assessing prediction potential related to MDR for isolates recovered from untreated and TE-treated media. Diagnostic performance using sensitivity and specificity for all bacterial isolates and *E. coli* isolates are presented in Table 6 and Table 7 for isolates from untreated media and TE-treated media, respectively. The corresponding areas under the receiver operating characteristic curve (AUCs) are summarized in Table 8. These results are organized for the isolates by untreated media and TE-treated media. The diagnostic statistics without stratification are viewable in Table S9.

Among all antibiotics assessed, AM non-susceptibility yielded the strongest diagnostic statistics. When focusing on all isolates, AM demonstrated 100% sensitivity and >91% specificity estimates, with an area under the ROC curve (AUC) of ≥96% when focusing on MDR prediction of recovered isolates from both untreated and TE-treated media. The next strongest diagnostic statistics were for cefazolin (CFZ) and ampicillin/sulbactam (A/S), which showed higher sensitivity (≥80%) and specificity (>90%) than the other antibiotics when focusing on *E. coli* from background conditions (no TE treatment). No antibiotics other than AM, CFZ, and A/S had AUC values > 80%.

For isolates recovered from TE-treated media, it was apparent that TE treatment successfully screened for TE non-susceptibility among isolates. Overall, TE non-susceptibility demonstrated relatively high sensitivity (87.5%) in predicting MDR while also exhibiting poor specificity (2.4%), resulting in a very low AUC (45%) when including all isolates recovered. For focusing the analysis on only the recovered *E. coli* isolates from the TE-treated media, due to complete separation, diagnostic statistics could not be determined. Overall, there were 53 *E. coli* isolates recovered from TE-treated media, of which 13 (24.5%) were MDR. All 53 isolates had non-susceptibility to TE, and 40 (75.5%) were not MDR. Therefore, TE non-susceptible *E. coli* isolates from the TE-treated media had 100% sensitivity for MDR but exhibited poor specificity.

## 3. Discussion

### 3.1. Antibiotic Non-Susceptibility and MDR Prevalence Among Recovered Isolates

Estimates of the true AMR prevalence among all *E. coli* from natural waters in this region are not achievable with this dataset, particularly due to the uneven, non-random, convenience water sampling approach and the limited isolate selection per plate. The study aimed to examine TE-non-susceptibility in TE-treated and untreated media but, in that process, uncovered an appreciable amount of MDR *E. coli*. Overall, 24% of all recovered isolates, which presented as presumptive *E. coli*, exhibited multidrug resistance (MDR). Upon performing species identification, and focusing only on *E. coli,* the prevalence of MDR dropped to 18%.

When stratifying for *E. coli* isolates with untreated media to better assess background levels of TE non-susceptibility, the prevalence of MDR *E. coli* dropped to 17% among the recovered *E. coli* isolates from ColiGlow (Table S12). One of the *E. coli* isolates obtained from Colilert was also MDR (Table S13). Inferences about MDR isolate recovery effectiveness of the different media types cannot be made from this limited dataset due to the uneven exploratory sampling approach used. It is important to note that all recovered isolates studied, including those originating from TE-treated ColiGlow and ColiGlow, were inoculated and grown in Colilert broth, where they all were presumptive *E. coli*, as they all changed the Colilert media color to yellow and fluoresced blue under longwave UV light.

While not yet well-described for this region of Kentucky, the finding of phenotypic AMR *E. coli* in Kentucky surface waters is not unexpected. Three proximal surface-water studies conducted within the past decade enable comparison of phenotypic antibiotic resistance: 496 isolates from the Upper Oconee River Watershed, Northeast Georgia (2015–2016) [34]; 329 isolates from the Maumee River, Northwest Ohio (2016–2017) [26]; and 177 isolates from Sinking Creek, Northeast Tennessee (2023) [35]. In the Maumee River study, 15% of isolates were resistant to more than three antibiotics and 1.2% to more than five antibiotics, levels that are similar to but higher than our findings when stratifying for untreated media. In contrast, MDR prevalence was lower in the Upper Oconee Watershed (3%) [34] and substantially higher in Sinking Creek, where 47.5% of isolates were resistant to more than three antibiotic categories and 5.1% to seven categories [35]. In comparison, 5 (3.9%) of our 128 *E. coli* isolates were non-susceptible to seven or more categories (Table 4).

Comparisons across studies are limited by differences in antibiotics tested and definitions of resistance and non-susceptibility as well as the uneven sampling approach employed here. The Maumee River study evaluated resistance to eight antibiotics, whereas this study assessed non-susceptibility to 21. Varying MDR definitions and antibiotic class coverage further limit comparisons, underscoring the need for standardized MDR definitions in One Health surveillance [36]. This study used the MicroScan system results to define MDR using the standardized definition of acquired non-susceptibility to at least one agent in three or more antimicrobial categories [33]. Despite these limitations, cross-study MDR and antibiotic-specific prevalence comparisons remain informative.

Ampicillin (AM) resistance was observed in 38.3% of isolates in the Maumee River study compared with 16% and 28% AM non-susceptibility among our Kentucky *E. coli* isolates from treated and untreated media, respectively. The Sinking Creek study did not evaluate AM resistance. The Oconee Watershed study had low resistance prevalence for all antibiotics and reported AM resistance as the second most common phenotype (2.2%), following TE resistance. Chesapeake Bay watershed studies associated with manure application reported 24% AM non-susceptibility, similar to our findings [37].

In our study, among the 77 *E. coli* isolates grown on untreated media to estimate background TE non-susceptibility, 18.2% were TE non-susceptible, which was higher (*p* = 0.012) than the 8.8% TE resistance reported for the Maumee River [26]. TE resistance was also common in Sinking Creek (30.5%) but less frequent in the Oconee Watershed (4.1%), where it remained the most prevalent resistance type [34,35]. TE resistance was frequently associated with MDR patterns in the Sinking Creek study.

Higher TE non-susceptibility in this study aligns with reports linking TE resistance to beef production globally [38,39,40]. A global meta-analysis estimated 31% TE non-susceptibility among *E. coli* isolates from beef cattle [38]. In Kentucky, tetracyclines—particularly oxytetracycline and chlortetracycline—are among the most used antibiotics in cattle [41,42], with bovine *E. coli* showing 56% TE non-susceptibility in 2017, down from 65% in 2012 [42]. Unlike the Maumee River watershed, which is dominated by row-crop agriculture [43], pastureland predominates in our study area (Figures S1–S3), increasing the likelihood of direct contamination from fresh manure. Fresh manure has been associated with higher abundances of antibiotic-resistant bacteria and resistance genes [44]. Runoff from historically grazed pasturelands has also been linked to elevated TE resistance [27].

### 3.2. Impact of Species Composition on Screeening Media for Characterizing MDR Prevalence

For researchers investigating AMR among aquatic *E. coli* using selective differential media, it is important to consider the true species composition of presumptive *E. coli* isolates. In the present study, MDR prevalence among presumptive *E. coli* isolates recovered from TE-treated and untreated media was 28% and 22%, respectively. After MALDI-TOF MS confirmation and exclusion of non-*E. coli* isolates, MDR prevalence decreased, although not significantly, to 25% and 13%, respectively (*p* = 0.957 and *p* = 0.058).

This reduction reflects the potential for higher MDR prevalence among non-target organisms recovered in the ColiGlow media. Members of the *Enterobacter cloacae* complex exhibited substantially higher MDR prevalence than *E. coli* (100% vs. 18%). *Enterobacter cloacae* complex and *Citrobacter* spp. are well recognized for possessing intrinsic β-lactam resistance and frequently exhibiting MDR phenotypes [33,45,46]. In addition, *Enterobacter cloacae* complex species may harbor the *uidA* gene [45,46], producing false-positive reactions on media designed for presumptive *E. coli* identification. Other genera, including *Citrobacter* and *Serratia*, may also be recovered and misclassified as *E. coli* without confirmatory species identification.

*Enterobacter cloacae* complex species have been reported to grow as presumptive *E. coli* on EPA-approved selective differential media, including mTEC and Colilert [47,48] and may fluoresce at 44.5 °C because of stable β-glucuronidase activity [49] and/or β-glucosidase activity [50]. Because β-glucosidase and β-glucuronidase can each generate fluorescent products from their respective substrates, fluorogenic or chromogenic assays targeting either enzyme remain susceptible to false-positive reactions from non-target organisms expressing the corresponding enzyme [51].

These findings have implications for studies incorporating antibiotics into selective media to estimate the prevalence or density of resistant *E. coli*. By design, antibiotic-supplemented media would preferentially recover antibiotic-resistant organisms for the antibiotic of interest. It is then plausible for non-target organisms with intrinsic resistance to be overrepresented, biasing estimates of MDR prevalence unless species identity is confirmed. For example, 18 of 21 (86%) non-*E. coli* isolates in this study were non-susceptible to ampicillin, suggesting that screening media supplemented with ampicillin could further increase the relative abundance of intrinsically resistant *Enterobacter* and *Citrobacter*, thereby inflating estimates of environmental MDR *E. coli* prevalence if AM-resistant isolates are selected for antibiotic susceptibility testing without species identification. While instruments like VITEK 2, Phoenix, and MicroScan systems perform phenotypic antimicrobial susceptibility testing as part of panels that simultaneously perform biochemical-based identification, not all methods for AMR determination are paired with advanced species identification methods, especially in limited resource regions where chromogenic media and *uidA* PCR may be used [49]. Recently, a multiplex PCR assay demonstrated strong potential for molecular AMR surveillance for *E. coli*; however, intrinsically resistant *Enterobacter cloacae* complex isolates complicated interpretation [50,52]. Species identification had previously occurred on separate panels prior to analysis.

An interesting secondary finding was that the relatively higher MDR prevalence observed among incidentally recovered aquatic *Enterobacter cloacae* complex isolates suggests these organisms may be useful targets for future environmental AMR surveillance consideration. Their incidental recovery indicates they may occur at appreciable densities in surface waters, and previous studies have reported novel resistance mechanisms among environmental *Enterobacter* spp. with potential clinical significance [53,54].

### 3.3. Sentinel Antibiotics for Predicting Culturable MDR and Their Potential Surveillance Value

#### 3.3.1. No Enhancement in MDR *E. coli* Isolate Recovery from Tetracycline in Media

Although the study design evaluated TE-treated media as a potential approach to screen for MDR *E. coli*, its performance was limited. TE treatment did not significantly increase recovery of MDR *E. coli* compared with untreated media, which served to characterize background resistance. Among the *E. coli* recovered from TE-treated media, all were TE non-susceptible, yet 24.5% were MDR, which was not significantly different from untreated media MDR *E. coli* isolate recovery (13.3%; *p* < 0.104). Although TE non-susceptibility achieved 100% sensitivity for MDR among recovered *E. coli*, it failed to distinguish MDR from non-MDR isolates, preventing estimation of specificity and overall discrimination because all isolates tested positive. In contrast, among *E. coli* recovered from untreated media, TE non-susceptibility demonstrated 50.0% sensitivity and 86.2% specificity (AUC = 0.68) for MDR classification, which is a lower discrimination statistic relative to other antibiotics examined. Future studies could evaluate whether combining TE with one or more additional antibiotics in selective *E. coli* media could improve the correct classification and recovery efficiency for MDR *E. coli* from Kentucky waters.

#### 3.3.2. Ampicillin and Cefazolin Non-Suceptiblity for Potential MDR Prediction

Among the most interesting findings from our analysis of MDR-associated antibiotic susceptibility profiles was the consistently high discrimination potential of AM and cefazolin (CFZ) non-susceptibility for identifying MDR isolates within this dataset, regardless of media (TE-treated or not). While diagnostic test selection requires more than discrimination alone [55], an AUC greater than 0.90 is generally considered excellent or outstanding [52,53,56], particularly when obtained from a separate validation dataset. In our study, the AUC values were obtained from the same (training) dataset and were not from a validation dataset; therefore, the AUCs may overestimate diagnostic performance and should be interpreted cautiously or as hypothesis-generating for future study.

Upon using AM non-susceptibility among recovered *E. coli* to correctly classify MDR, the AUC values were ≥0.96 for the classification of MDR *E. coli* isolates from TE-treated and/or untreated media. Similarly, CFZ non-susceptibility demonstrated relatively high AUC values (≥0.85) for both media types. These findings suggest that AM and/or CFZ non-susceptibility may serve as potential sentinel markers for MDR *E. coli* and are therefore the strongest candidates among the antibiotics assessed for potential incorporation into *E. coli* media, aiming to increase the recovery efficiency of MDR *E. coli* isolates from natural waters in this region of Kentucky.

The association between AM non-susceptibility and MDR has been reported previously [54]. Although not observed here, one limitation of AM as a potential sentinel marker in some settings is its high prevalence of non-susceptibility, occurring in more than 70% of environmental isolates [57,58,59] and over 50% of clinical isolates [60], with even higher rates reported in resource-limited regions [61,62,63]. In such contexts, widespread AM non-susceptibility could reduce specificity for identifying MDR. However, in rural or less disturbed watersheds, where AM non-susceptibility may be less common, it may retain greater utility as a screening marker. CFZ also demonstrated strong discrimination in this study, consistent with prior work identifying CFZ use as strongly associated with antibiotic-resistant *E. coli* infections [64]. As with AM, however, the usefulness of CFZ as a sentinel marker may diminish in settings where CFZ non-susceptibility is highly prevalent, as suggested by global clinical data reporting 74% non-susceptibility among *E. coli* isolates [65].

### 3.4. Recovery of Potential Extensively Drug-Resistant Isolate and Environmental Context

The potential XDR isolate was obtained from White Oak Creek in Estill County, Kentucky. Definitive classification of this isolate as XDR was not possible because the MicroScan panels used did not assess non-susceptibility for 4 of the 16 antimicrobial categories included in the definition of Magiorakos et al. [33]: ceftaroline, chloramphenicol, fosfomycin, and polymyxins (represented by colistin). These agents are not routinely included in automated antimicrobial susceptibility testing (AST) panels for *Enterobacteriaceae* because of limited clinical applicability (e.g., chloramphenicol is rarely prescribed due to toxicity [66]) and the technical limitations of measuring non-susceptiblity to fosfomycin and colistin via microdilution [67,68].

The recovered *E. coli* isolate from TE-treated media was from a predominantly deciduous forest watershed near a small population center (~2000 residents). The StreamStats database of the U.S. Geological Survey indicates that the catchment area above the sample collection point is 17.74 km^2^, of which 0.836% or 0.15 km^2^ is impervious [69]. The terrain is rugged, characteristic of the Appalachian foothills, with catchment area elevations ranging from 304 m above sea level to low areas below 228 m (Figure S7). Proximal to the sampled point is a regional solid waste landfill, active since 1984, located 1500 m north of the sample collection point, which has a tributary to the same stream, but the confluence is downstream of the collection point (Figure S8). The land uses in the areas immediately adjacent to the stream are predominantly pasture/hay, comingled with developed areas for residential/agricultural buildings and roadways that run parallel to the stream and tributaries (Figure S9). The environmental characteristics of this watershed provide context that potential XDR isolates can be recovered in mostly rural areas with some disturbance or development, but conclusions regarding the influence of specific land uses or contamination cannot be made on the occurrence of this *E. coli* isolate.

### 3.5. Future Research Opportunities Using Molecular Approaches

Beyond the culturable surveillance approach used in our study, molecular approaches aimed at identifying the presence/absence of known resistance genes would enhance our understanding of the potential for the environment to present novel genes and/or unknown mechanisms involved in aquatic- or environment-associated AMR. Additionally, molecular approaches could elucidate which known genes are most dominant and responsible for the observed non-susceptibility to antibiotics, such as *mdtK*, *macB*, and *ampC*, which have been linked to MDR among the isolates of *E. coli* [70]. Routine screening on isolates demonstrating MDR and carbapenem and/or beta-lactam non-susceptibility could be performed using established panels to understand the clinical implications of these isolates relative to known treatments. Notably, assays or panels used in clinical environments, such as the NG-Test CARBA 5 (Guipry, France), BioFire^®^ FilmArray (Salt Lake City, UT, USA), Cepheid GeneXpert^®^ (Sunnyvale, CA, USA), and Roche Diagnostics cobas^®^ eplex (Carlsbad, CA, USA), can simultaneously assess isolates for several known carbapenem resistance genes (*bla*_IMP_, *bla*_KPC_, *bla*_OXA-48_, *bla*_NDM-_, *bla*_VIM_) and ESBL-production (*bla* CTX-M) genes; unusual resistance not explained by genes (negative gene tests) may have implications on research needs or increased surveillance.

A strength of a tandem approach (culture-based and molecular) is that sequence-based methods rely on known antibiotic resistance genes (ARGs) and can thus miss novel determinants of clinically relevant phenotypic resistance. Studies using functional metagenomics have observed considerable diversity of ARGs in the environment, particularly in the soil, including novel genes for previously undescribed β-lactamases; however, these methods have difficulty in identifying the host microorganism(s) [71,72,73]. Lessons learned from metagenomics indicate the microbial communities have many other strategies for resisting antibiotics than are known, and understanding phenotypic non-suspetibility to clinical antibiotics continues to be of significance for clinical practice as well as understanding emerging AMR threats.

As resistance genes can circulate among diverse bacteria via horizontal gene transfer and can persist even without direct selection pressure, wildlife and natural environments remain understudied in the broader resistance landscape as intersecting with human and animal health. Given these dynamics, comprehensive AMR surveillance can use approaches that use culture- and molecular-based approaches. Within this broader framework, our focus was surface water-oriented surveillance aimed at a monitoring approach for identifying potential sentinel antibiotics to incorporate into media for recovering MDR *E. coli* isolates efficiently. The approach demonstrated feasibility as a strategy for recovering MDR *E. coli* isolates, and further research, including validation studies involving proposed media supplementation with AM or CFZ, may be useful for incorporation into One Health-oriented surveillance frameworks that monitor antibiotic-specific and multidrug resistance trends, link environmental AMR signals to fecal contamination, and identify potential resistance hotspots [7,11,24,25,30,74,75].

### 3.6. Study Limitations

Several study limitations impact study interpretations and generalizability. The most significant limitations relate to the (1) convenience sampling approach, (2) environmental covariates not being assessed, (3) very limited isolate selection, (4) potential biases that may be associated with the selective differential media used, and a (5) lack of molecular characterization of mobile genetic elements and resistance genes. Additionally, limitations relate to the uncertainty over the MIC values to use with environmental *E. coli* isolates for determining non-susceptibility to individual antibiotics as well as whether clinical MIC values are appropriate.

While efforts were made to sample a cross-section of headwater streams in the study area, several locations were sampled more than once, and most samples were obtained from Madison County, relative to the other counties. In Madison County, nearly all publicly accessible streams with water were assessed; however, many first-order streams were not assessed due to inaccessibility or a lack of water due to dry weather. The uneven sampling does not enable the prevalence of individual antibiotic non-susceptibilities and MDR among recovered isolates to be generalized to the region. Despite isolates from TE-treated and untreated media coming from the same grab sample, these isolates were treated as independent observations. Furthermore, while several sites were revisited, the isolates selected from these sites were also treated independently. The analysis not accounting for clustering or a lack of independence is an additional limitation impacting generalizability.

Regarding environmental conditions, heavy rainfall events may have been linked to higher frequencies of antibiotic resistance as well as MDR prevalence. In a global meta-analysis, dry regions of the world, which can experience more AMR, showed elevated levels of antibiotic resistance among *E. coli* during extreme rain events, ostensibly due to do flushing and an overall increase in fecal contamination [76]. Many studies have demonstrated an increase in antibiotic resistance genes and/or mobile genetic elements in aquatic systems following rainfall events [77,78,79,80,81,82,83]; however, some research has observed an increase in fecal indicator bacteria without an increase in resistant strains [83]. Stormwater releases, which increase during rainfall, could also contribute to AMR given that a high prevalence of AMR genes has been observed in stormwater [84,85]. Future studies using retrospective rainfall and land use data could be used to examine their importance for predicting patterns of non-susceptibility to individual antibiotics and the prevalence of MDR.

Overall, the study examined 151 isolates presenting as magenta- or red-colored colonies on modified mTEC agar. The typical selection of one colony per plate, and uneven periodic selection of two colonies, limits the generalizability of the prevalence of non-susceptibility and MDR among recovered isolates. While several unique colony morphologies could be evaluated per plate, it is possible that typical colony morphology was more likely to be selected. By selecting several colonies with typical and atypical morphology, a more representative characterization of the MDR *E. coli* in this region could have been obtained. For example, smaller colonies may have been less likely to be selected but could possess a different non-susceptibility profile than larger or typical colonies. Also, using differential media with a specific chromogen, it is possible that β-glucuronidase-negative *E. coli*, which evaded detection in our study approach, may have a different prevalence of phenotypic non-susceptibility than the β-glucuronidase-positive (magenta) isolates on the mTEC plates. Previous research on β-glucuronidase-negative colonies have linked some to the pathogenic, often cattle-associated, STEC O157 serotype, which had less ESBL-producing isolates and possibly lower prevalence of MDR or AMR—hypothesized as potentially due to human infections from O157 typically not being treated with antibiotics and thereby removing selection pressure on this serotype [86].

Lastly, there were 23 MDR *E. coli* isolates displaying phenotypic MDR based on clinical breakpoints for isolates that have been encountered in clinical environments. The application of clinical MIC breakpoints aimed at treating human disease may not be appropriate for environmental isolates [49,87] but, in the absence of established breakpoints, do assist in providing some context. It is unknown from this study the mechanisms enabling these isolates to be non-susceptible. If the study included multiplex PCR examining genes related to the production of β-lactamases, including, but not limited to, genes specific to resisting carbapenems and ESBL antibiotics, such as *bla*_CTX-M_, *bla*_TEM_, *bla*_KPC_, *bla*_NDM-1_, *bla*_OXA_, *bla*_VIM_, and *bla*_AmpC_, then there would be greater evidence supporting the phenotypic non-susceptibility being due to the organism and not the research conditions. Furthermore, repeated measures of phenotypic resistance where no known molecular mechanism was observed would have warranted further research to potentially uncover new mechanisms not able to be discovered using a solely molecular-based study design.

## 4. Materials and Methods

### 4.1. Water Sample Collection

The isolates evaluated in this study were recovered from 96-well plates and Idexx Quanti-Trays aimed at enumerating the most probable number (MPN) of *E. coli* and presumptive TE-resistant *E. coli* from water samples in East-Central Kentucky. The isolates were obtained from water samples collected in June and July of 2022 and 2024 from Madison, Estill, Garrard, Powell, and Rowan Counties in Kentucky (Table 1). For obtaining a range of *E. coli* densities, samples were obtained in dry and wet weather and included sites with dominant land uses including urban residential, pasture/grazing lands, and limited agricultural development. Additionally, sites with >95% forestland (e.g., Owsley Fork [Madison County], Daniel Boone National Forest [Estill, Powell, and Rowan County]) were selected. The convenience sampling approach emphasized the exploration of many small streams to enhance discovery and land use diversity, aimed at guiding future hypotheses; this limited the generalizability of the results and prevalence estimates related to antibiotic susceptibility. In all water sampling efforts, samples were collected in 1.5 L sterile Nalgene sampling containers with sufficient water to enable split sampling to enumerate *E. coli* densities in media with and without tetracycline. All water samples were collected in a manner so as to not disturb the sediment. From these water samples, resources were available for a limited amount of antibiotic susceptibility testing (AST). AST was performed on 30 isolates obtained from 18 of the 28 locations in 2022 and for 121 isolates obtained from 32 different locations in 2024.

### 4.2. Water Quality Determination by E. coli Enumeration

Each water sample was analyzed using the ColiGlow method with and without tetracycline, which is an MPN-based method using a 96-well plate for each sample. Each water sample in 2024 was also analyzed using the Idexx Colilert [9] MPN method. Periodic negative control samples (approximately one per 30 water samples) were tested using sterile distilled water for the three methods to ensure no background contamination.

For the ColiGlow method, 22.5 mL of sample was added to sterile 50 mL tubes containing either 2.5 mL of the selective differential growth media or 2.5 mL of the same media supplemented (treated) with 640 µg of TE to enable the quantification of all *E. coli* and TE-resistant *E. coli*, respectively. After mixing samples with media (using at least 30 inversions of the vial containing the sample and media), the mixtures were then dispensed into 96-well plates (200 µL per well) with a multichannel pipette and incubated at 37 °C for 24 h. Fluorescing wells observed under longwave UV light after 24 h were counted as presumably positive for *E. coli* based on β-D-glucuronidase cleavage of MUG to the fluorescent compound 4-MU. The MPN per 100 mL was estimated using the method’s MPN table, with a detection range of 14–1479 MPN/100 mL, where one positive well was equivalent to 14 MPN per 100 mL, no positive wells were reported as <14, and fully positive plates (all 96 wells fluorescing) were >1479 MPN/100 mL. On days with considerable wet weather (rainfall), a 10% dilution was used to enable a modification of the enumeration range from 140 to 14,790 MPN/100 mL. As samples were analyzed in a paired approach using media with and without TE, the density of total *E. coli* and TE-resistant *E. coli* was able to be ascertained in MPN/100 mL as described in our previous study [19].

For the Idexx Colilert method, 100 mL of sample was added to a sterile vial and mixed with Colilert powdered media using 30 inversions. The mixture was then poured into a single-use foil-backed Quanti-Tray 2000 and sealed using a Quanti-Tray sealer. The sealed Quanti-Tray containing the media was incubated at 37 °C for 24 h. Fluorescing wells observed under longwave UV light after 24 h were counted as presumably positive for *E. coli*. The MPN per 100 mL was recorded using the method’s MPN table, with a detection range of 1–2419.6 MPN/100 mL.

### 4.3. Isolate Selection

Fluorescing wells from the 96-well plates were used for enumerating microbiological water quality and, along with Colilert quanti-trays, were used in the isolate recovery process. Of greatest interest were 96-well plate pairs where there was growth in tetracycline-treated and untreated media, for which one randomly selected well with evidence of positive *E. coli* growth in media with and without tetracycline would be selected to compare TE-resistant and/or non-susceptibility recovery between media types. There were several samples that had no fluorescent wells on the tetracycline-treated plates (indicative of no tetracycline-resistant *E. coli*) but produced growth in the media not supplemented with tetracycline, used for assessing background conditions when there was no evidence of TE resistance. In these cases, one well from the media with growth was randomly selected for isolate recovery. Similarly, there were randomly selected quanti-trays with growth to further assess background conditions when TE was not within the media.

Using sterile 10 µL disposable loops, broth contained in the selected wells with growth was inoculated onto 100 mm Petri dishes containing m-TEC ChromoSelect agar (Millipore Sigma, Catalog No. 90924, Darmstadt, Germany), a standard media used as part of EPA Method 1603 [8]. A similar method was employed using randomly selected Colilert trays with growth after creating an opening in the foil-backed Quanti-Tray using flame-sterilized beveled forceps to remove 10 µL of inoculum.

Incubation on mTEC was performed for 24 h at 37 °C, and following incubation, colony morphology was examined. Often, colonies with presumptive *E. coli* color (purple-red, magenta, and/or maroon) had similar morphology on the plate with respect to shape, size, etc. In cases where there were color or shape differences between isolates from samples in 2022, the isolates that resembled typical *E. coli* appearance were selected. The isolates from mTEC agar were then inoculated into 1.0 mL of Idexx Colilert broth to re-assess their likelihood of being *E. coli* by β-D-glucuronidase cleavage of MUG to the fluorescent compound 4-MU, as well as their ability to also use Idexx Colilert media through their β-galactosidase activity, which hydrolyzes ONPG to produce the yellow compound o-nitrophenol. The Colilert broth was created using 100 mL of sterile distilled water with one packet of Colilert media, which was then distributed into 1.5 mL centrifuge tubes. The isolates that were confirmed by the Idexx Colilert media as presumptive *E. coli* were then prepared for confirmatory identification and AST.

### 4.4. Antibiotic Susceptibility Testing (AST) and Species Identification

For AST and biochemical-based identification, the MicroScan autoSCAN-4 (Beckman Coulter; Brea, CA, USA) instrument was used. Isolates from the Colilert broth were plated onto blood agar plates and incubated for 24 h at 36 °C. Pure isolates from the blood agar plates were then inoculated and incubated on MicroScan Negative Urine Combo 85 panels (Beckman Coulter, Catalog No. B1017-435) in accordance with the manufacturer’s instructions. Panels were read by the autoSCAN-4 system utilizing the LabPro v.4.42 database. AST results were interpreted in accordance with clinical breakpoints, as specified in Table 9. For isolates exhibiting resistance to five or more categories or isolates displaying unusual resistance, the test was repeated for confirmation.

All isolates from 2024 were evaluated with MALDI-TOF-MS for subsequent species identification testing using the VITEK MS Prime (bioMérieux; Marcy-l’Étoile, France) system with knowledge base v3.2 and RUO Saramis knowledge base v4.17. Repeat identification testing was performed on isolates from which no identification or low confidence scores were obtained on initial testing with both instruments.

### 4.5. Maps and County-Specific Land Use Figures

County-specific land use maps, available in the Supplementary Materials, were accessed, and images were captured through the U.S. Multi-Resolution Land Characteristics (MRLC) Consortium via the National Land Cover Database viewer [88]. The maps labeling the locations where MDR isolates were recovered in the Supplementary Tables and the study area map (Figure S6) were generated in RStudio via the open-source data science company Posit [89]. ChatGPT [90] was used for assisting in code generation for designing the maps in RStudio. For placement of MDR isolate recovery points on maps, latitude and longitude values for sample locations and nearby communities were provided to ChatGPT to generate the RStudio code. Additional editing of the code was done within RStudio to adjust legends. The maps were then accessed for accuracy and deemed consistent with observations.

### 4.6. Statistical Analysis and Defining Multidrug Resistance

All statistical analyses were performed in Stata 14.2, including the hypothesis-based tests and acquisition of diagnostic statistics, with the dataset available in the Supplementary Materials (Supplementary File S2). For evaluating the treatment effect of adding TE to the media, a paired analysis was performed examining the microbiological water quality of each isolate using the density of presumed *E. coli* MPN per 100 mL and presumed TE-non-susceptible *E. coli* MPN per 100 mL. Results exceeding the method range (>1479 if no dilution was used or >14,790 if a 10% dilution was used) were set as 1479 or 14,790 MPN per 100 mL to enable a nonparametric analysis using the Wilcoxon signed-rank test. Hypothesis-based tests using binary-based classifications of microbiological water quality (frequency of over-range results versus in-range results) were performed using chi-square analysis.

Non-susceptibility to each antibiotic was defined as a binary term (0 = Susceptible, 1 = NS) for each isolate evaluated, where NS (not susceptible) was defined as having a minimum inhibitory concentration (MIC) at either the intermediate or resistant threshold level (Table 9). This binary classification was then used for determining MDR.

MDR was defined as a binary term (0 = NS to <3 categories of antibiotics, 1 = NS ≥ 3 categories) using the definition of Magiorakos et al. [33] (non-susceptible to ≥1 agent in ≥3 antimicrobial categories). There was a total of nine categories of antibiotics for the MDR calculation, defined as follows: (1) penicillins, β-lactam, β-lactamase inhibitor combinations; (2) cephalosporins; (3) carbapenems; (4) monobactams; (5) aminoglycosides; (6) fluoroquinolones; (7) folate pathway inhibitors; (8) tetracyclines; and (9) nitrofurans. Using binary classifications for susceptibility and NS, as well as susceptible, intermediate resistance, and resistance, stacked bar charts were generated in Stata to act as antibiograms of the isolates studied. Potential XDR was defined as NS for all but two antibiotic categories of the nine categories assessed. Magiorakos et al. [33] defines XDR for *Enterobacteriaceae* as NS for all but 2 categories of 17.

Analyses comparing the prevalence of non-susceptibility or MDR between groups were performed using many individual chi-square and exact tests. Chi-square tests were used when all four cell counts in 2 × 2 tables produced values of five or higher. In 2 × 2 tables containing cell counts with less than five observations, including zero, Fisher’s exact tests were performed. Given the multiple comparisons, a Holm–Bonferroni correction was used to adjust *p*-values obtained from chi-square and Fisher’s exact tests. Significance was determined as *p* < 0.05 after adjustment for multiple comparisons. Statistical analyses did not account for clustering or the lack of independence for isolates originating from the same sampling location or in cases where a second isolate was recovered from a different positive well on the same 96-well plate.

Diagnostic statistics were obtained to assess the predictive ability of NS for each antibiotic for predicting MDR. First, the “diagt” package from the Statistical Software Components archive was installed in Stata via the command “ssc install diagt”. Subsequently, the diagnostic statistics of sensitivity, specificity, positive predictive value, and negative predictive value were obtained using the “diagt” command, which provided the associated Exact (Clopper-Pearson) 95% confidence intervals and AUC. Due to complete separation (scenarios where there was total susceptibility or non-susceptibility among all isolates), some diagnostic statistics were not able to be calculated or estimated.

## 5. Conclusions

The prevalence of MDR among recovered *E. coli* isolates from natural water samples collected in East-Central Kentucky, U.S., in 2022 and 2024 was 13% in media without TE. While the MDR prevalence was similar to a study from nearby Ohio, U.S., which had 15% prevalence for MDR *E. coli* isolates, the true prevalence of MDR isolates in this region of Kentucky was not able to be determined due to the convenience sampling approach used and limited number of isolated colonies examined per sample, all of which limit generalizability. An observation useful for studies using an enzyme substrate method for identifying or enumerating *E. coli* isolates is that incidentally recovered *Enterobacter* spp., which present like *E. coli*, had markedly higher MDR prevalence, confirming results from other studies focused on *Enterobacter* spp. Therefore, studies may overestimate MDR and/or non-susceptibility to individual antibiotics among *E. coli* if there are *Enterobacter* spp. in samples, since some *Enterobacter* spp. appear like *E. coli* and are more likely to grow in the presence of antibiotics.

For efforts aimed at identifying candidate antibiotics for use in MDR screening media for environmental *E. coli*, isolates with AM and CFZ non-susceptibility were most associated with MDR in this dataset. Studies using AM- and/or CFZ-supplemented media would be needed to determine their predictive potential for MDR isolate recovery in practice. While contrary to the study hypothesis, TE supplementation had poor specificity for identifying MDR isolates in this convenience sample. Overall, this study demonstrated the potential of culture-based techniques coupled with AST to recover aquatic MDR *E. coli* isolates and one potentially XDR isolate. Future studies incorporating AM and/or CFZ into selective differential *E. coli* media, possibly in tandem with TE, could resolve the specificity problem for recovering environmental MDR *E. coli* isolates, which may be of interest for monitoring or studying their mechanisms of antibiotic resistance.

## Figures and Tables

**Table 1:** Number of unique sampling sites associated with the 151 isolates recovered from ColiGlow, Colilert, and tetracycline-supplemented ColiGlow media, organized by year and county, 2022–2024.

Year	County	Unique Sites	Isolates from Media Without TE		Isolates from Media with TE
			ColiGlow (No. of Sites)	Colilert (No. of Sites)	ColiGlow with TE (No. of Sites)
2022	Madison	7	10 (6)	0 (0)	12 (7)
2022	Powell	3	4 (3)	0 (0)	1 (1)
2022	Rowan	2	2 (2)	0 (0)	1 (1)
2022	2022 Sum	12	16 (11)	0 (0)	14 (9)
2024	Madison	34	53 (25)	20 (16)	38 (17)
2024	Estill	3	0	0 (0)	4 (3)
2024	Garrard	2	1 (1)	3 (2)	2 (1)
2024	2024 Sum	39	54 (26)	23 (18)	44 (21)
2022–24	Combined	47	70 (35)	23 (18)	58 (29)

**Table 2:** Distribution of *E. coli* densities (most probable number [MPN]/100 mL) and tetracycline (TE)-resistant *E. coli* densities for the water samples used for obtaining the bacterial isolates studied.

Isolate Group		*E. coli* MPN per 100 mL		
	N	25th	Median	75th
All isolates (including *E. coli* and non-*E. coli*)	151	119	959	≥1479
All *E. coli* isolates	130	119	890	≥1479
All non-*E. coli* isolates	21	89	≥1479	≥1479
All untreated media isolates (including *E. coli* and non-*E. coli*)	93	89	705	≥1479
*E. coli* isolates from untreated media	77	89	291	≥1479
Non-*E. coli* isolates from untreated media	16	179	≥1479	≥1479
All isolates from TE-treated media (including *E. coli* and non-*E. coli*)	58	451	≥1479	≥1479
*E. coli* isolates from TE-treated media	53	451	≥1479	≥1479
Non-*E. coli* isolates from TE-treated media	5	72	≥1479	≥1479
		**TE-Resistant** ***E. coli* MPN per 100 mL**		
All isolates from TE-treated media (including *E. coli* and non-*E. coli*)	58	33	77.5	227
*E. coli* isolates from TE-treated media	53	39	80	394
Non-*E. coli* isolates from TE-treated media	5	14	66	123

**Table 3:** Isolates with discordant species identification using the Beckman Coulter MicroScan system and MALDI-TOF mass spectrometry (VITEK^®^ MS PRIME), with corresponding multidrug resistance (MDR) classification.

MicroScan Species ID (No. of Occurrences)	MALDI-TOF-MS Species ID	Multidrug Resistant ^1^ (Yes/No)
*Citrobacter farmeri* (x2)	*E. coli*	No
*Citrobacter freundii*	*E. coli*	No
*Enterobacter cloacae*	*E. coli*	Yes
*E. coli*	*Klebsiella aerogenes*	No
*E. coli*	*Klebsiella pneumoniae*	No
*E. coli*	Unknown	Yes
*Klebsiella pneumoniae*	*E. coli*	No
*Kluyvera ascorbata*	*E. coli*	No
*Morganella morganii*	*E. coli*	Yes
*Salmonella enterica* (x2)	*E. coli*	Not Determined ^2^

^1^ MDR was defined as non-susceptibility to at least one antimicrobial agent in three or more antimicrobial categories. ^2^ Antibiotic susceptibility was not determined for all antibiotic categories for *S. enterica* when identified by MicroScan as *S. enterica*; therefore, MDR was not determined.

**Table 4:** Bacterial species observed among 149 selected isolates and their frequency of non-susceptibility to 13 antibiotic categories for determining multidrug resistance (MDR); MDR is defined as non-susceptibility to at least one antibiotic in three or more antibiotic categories.

Organism ID	No.	No. of Antibiotic Categories with Non-Susceptibility											No. (%MDR)
		0	1	2	3	4	5	6	7	8	9	10	
*Citrobacter* sp.	1	-	-	-	-	-	1	-	-	-	-	-	1 (100.00)
*E. coli*	128	51	49	5	5	12	1	-	1	3	-	1	23 (17.97)
*Enterobacter* sp.	9	-	-	-	1	2	4	2	-	-	-	-	9 (100.00)
*Klebsiella* sp.	7	1	4	1	-	-	1	-	-	-	-	-	1 (14.29)
*Kluyvera* sp.	2	*-*	-	2	-	-	-	-	-	-	-	-	0 (0.00)
*Serratia* sp.	2	*-*	-	-	-	1	-	1	-	-	-	-	2 (100.00)
Total	149	52	53	8	6	15	7	3	1	3	0	1	36 (24.16)

**Table 5:** The frequency of multidrug-resistant isolates presented across three ordinal levels of microbiological water quality (EC-1, EC-2, and EC-3) for the *E. coli* isolates and all isolates as stratified by media without tetracycline and media with tetracycline. EC1: <100 *E. coli* per 100 mL, EC2: 100 ≤ n < 1479 *E. coli* per 100 mL, EC3: ≥1479 *E. coli* per 100 mL.

Microbiological Water Quality Categories	Species ID Isolate Type	Isolates from Media Without TE		Isolates from TE-Treated Media	
		No.	No. (% MDR)	No.	No. (% MDR)
EC-1: <100 MPN/100 mL	*E. coli*	22	5 (22.7%)	6	3 (50%)
EC-2: 100–1479 MPN/100 mL	*E. coli*	34	4 (11.8%)	17	3 (17.7%)
EC-3: ≥1479 MPN/100 mL	*E. coli*	19	1 (5.3%)	30	7 (23.3%)
Summary Total	*E. coli*	75	10 (13.3%)	53	13 (24.5%)
EC-1: <100 MPN/100 mL	All Isolates	26	6 (23.1%)	8	4 (50%)
EC-2: 100–1479 MPN/100 mL	All Isolates	36	6 (16.7%)	17	3 (17.7%)
EC-3: ≥1479 MPN/100 mL	All Isolates	29	8 (27.6%)	33	9 (27.3%)
Summary Total	All Isolates	91	20 (22.0%)	58	16 (27.6%)

**Table 6:** The sensitivity and specificity for characterizing the diagnostic performance of individual antibiotic non-susceptibility (NS) for predicting multidrug resistance (NS ≥ 3 categories) among all isolates (*n* = 91) and *E. coli* only (*n* = 75) isolates recovered from untreated media.

Class	Antibiotic	All Isolates (*n* = 91) Untreated Media		*E. coli* Isolates Only (*n* = 75) Untreated Media	
		Sensitivity % (95% CI)	Specificity % (95% CI)	Sensitivity % (95% CI)	Specificity % (95% CI)
Penicillins	AM	100.0 (83.2–100.0)	91.5 (82.5–96.8)	100.0 (69.2–100.0)	96.9 (89.3–99.6)
β-Lactam/ β-Lactamase Inhib. Combo	A/S	80.0 (56.3–94.3)	98.6 (92.4–100.0)	70.0 (34.8–93.3)	98.5 (91.7–100.0)
	AUG	75.0 (50.9–91.3)	100.0 (94.9–100.0)	60.0 (26.2–87.8)	100.0 (94.5–100.0)
	P/T	-	-	-	-
1st CEP	CFZ	85.0 (62.1–96.8)	98.6 (92.4–100.0)	80.0 (44.4–97.5)	98.5 (91.7–100.0)
2nd CEP	CTN	50.0 (27.2–72.8)	100.0 (94.9–100.0)	20.0 (2.5–55.6)	100.0 (94.5–100.0)
3rd CEP	CAX	10.0 (1.2–31.7)	100.0 (94.9–100.0)	10.0 (0.3–44.5)	100.0 (94.5–100.0)
	CAZ	30.0 (11.9–54.3)	100.0 (94.9–100.0)	30.0 (6.7–65.2)	100.0 (94.5–100.0)
	CFT	-	-	-	-
4th CEP	CPE	-	-	-	-
Carbapenems	ETP	-	-	-	-
	MER	0.0 (0.0–16.8)	98.6 (92.4–100.0)	0.0 (0.0–30.8)	98.5 (91.7–100.0)
Monobactams	AZT	-	-	-	-
Amino- glycosides	AK	-	-	-	-
	GM	-	-	-	-
	TO	5.0 (0.1–24.9)	100.0 (94.9–100.0)	10.0 (0.3–44.5)	100.0 (94.5–100.0)
Fluoroquinolones	CP	20.0 (5.7–43.7)	98.6 (92.4–100.0)	10.0 (0.3–44.5)	100.0 (94.5–100.0)
	LVX	15.0 (3.2–37.9)	100.0 (94.9–100.0)	10.0 (0.3–44.5)	100.0 (94.5–100.0)
Folate Pathway Inhibitor	T/S	5.0 (0.1–24.9)	100.0 (94.9–100.0)	10.0 (0.3–44.5)	100.0 (94.5–100.0)
Tetracycline	TE	55.0 (31.5–76.9)	83.1 (72.3–91.0)	50.0 (18.7–81.3)	86.2 (75.3–93.5)
Nitrofurans	FD	45.0 (23.1–68.5)	97.2 (90.2–99.7)	20.0 (2.5–55.6)	98.5 (91.7–100.0)

A/S: ampicillin–sulbactam, AK: amikacin, AM: ampicillin, AUG: amoxicillin–clavulanic acid, AZT: aztreonam, CP: ciprofloxacin, ETP: ertapenem, FD: nitrofurantoin, GM: gentamicin, LVX: levofloxacin, MER: meropenem, P/T: piperacillin–tazobactam, T/S: trimethoprim–sulfamethoxazole, TE: tetracycline, TO: tobramycin. CFZ: cefazolin, CTN: cefotetan, CAX: ceftriaxone, CAZ: ceftazidime, CFT: cefotaxime, CPE: cefepime.

**Table 7:** The sensitivity and specificity for characterizing the diagnostic performance of individual antibiotic non-susceptibility (NS) for predicting multidrug resistance (NS ≥ 3 categories) using all isolates (*n* = 58) and the *E. coli* only (*n* = 53) isolates recovered from media containing TE.

Class	Antibiotic	All Isolates (*n* = 58) TE-Treated Media		*E. coli* Only Isolates (*n* = 53) TE-Treated Media	
		Sensitivity % (95% CI)	Specificity % (95% CI)	Sensitivity % (95% CI)	Specificity % (95% CI)
Penicillins	AM	100.0 (79.4–100.0)	92.9 (80.5–98.5)	100.0 (75.3–100.0)	95.0 (83.1–99.4)
β-Lactam/ β-Lactamase Inhib. Combo	A/S	75.0 (47.6–92.7)	100.0 (91.6–100.0)	69.2 (38.6–90.9)	100.0 (91.2–100.0)
	AUG	31.2 (11.0–58.7)	100.0 (91.6–100.0)	15.4 (1.9–45.4)	100.0 (91.2–100.0)
	P/T	-	-	-	-
1st CEP	CFZ	75.0 (47.6–92.7)	100.0 (91.6–100.0)	69.2 (38.6–90.9)	100.0 (91.2–100.0)
2nd CEP	CTN	18.8 (4.0–45.6)	100.0 (91.6–100.0)	7.7 (0.2–36.0)	100.0 (91.2–100.0)
3rd CEP	CAX	37.5 (15.2–64.6)	100.0 (91.6–100.0)	38.5 (13.9–68.4)	100.0 (91.2–100.0)
	CAZ	50.0 (24.7–75.3)	100.0 (91.6–100.0)	46.2 (19.2–74.9)	100.0 (91.2–100.0)
	CFT	31.2 (11.0–58.7)	100.0 (91.6–100.0)	38.5 (13.9–68.4)	100.0 (91.2–100.0)
4th CEP	CPE	31.2 (11.0–58.7)	100.0 (91.6–100.0)	38.5 (13.9–68.4)	100.0 (91.2–100.0)
Carbapenems	ETP	6.2 (0.2–30.2)	97.6 (87.4–99.9)	7.7 (0.2–36.0)	97.5 (86.8–99.9)
	MER	-	-	-	-
Monobactams	AZT	31.2 (11.0–58.7)	100.0 (91.6–100.0)	38.5 (13.9–68.4)	100.0 (91.2–100.0)
Amino- glycosides	AK	-	-	-	-
	GM	6.2 (0.2–30.2)	100.0 (91.6–100.0)	7.7 (0.2–36.0)	100.0 (91.2–100.0)
	TO	6.2 (0.2–30.2)	100.0 (91.6–100.0)	7.7 (0.2–36.0)	100.0 (91.2–100.0)
Fluoroquinolones	CP	31.2 (11.0–58.7)	95.2 (83.8–99.4)	38.5 (13.9–68.4)	95.0 (83.1–99.4)
	LVX	31.2 (11.0–58.7)	97.6 (87.4–99.9)	38.5 (13.9–68.4)	97.5 (86.8–99.9)
Folate Pathway Inhibitor	T/S	43.8 (19.8–70.1)	100.0 (91.6–100.0)	46.2 (19.2–74.9)	100.0 (91.2–100.0)
Tetracycline	TE	87.5 (61.7–98.4)	2.4 (0.1–12.6)	-	-
Nitrofurans	FD	6.2 (0.2–30.2)	100.0 (91.6–100.0)	-	-

A/S: ampicillin–sulbactam, AK: amikacin, AM: ampicillin, AUG: amoxicillin–clavulanic acid, AZT: aztreonam, CP: ciprofloxacin, ETP: ertapenem, FD: nitrofurantoin, GM: gentamicin, LVX: levofloxacin, MER: meropenem, P/T: piperacillin–tazobactam, T/S: trimethoprim–sulfamethoxazole, TE: tetracycline, TO: tobramycin. CFZ: cefazolin, CTN: cefotetan, CAX: ceftriaxone, CAZ: ceftazidime, CFT: cefotaxime, CPE: cefepime.

**Table 8:** Areas under the receiver operating characteristic curve (AUCs) for assessing the discrimination potential of individual antibiotic susceptibility test results to predict the recovery of phenotypic multidrug-resistant (MDR) *E. coli* isolates and MDR isolates appearing like *E. coli*.

Class	Antibiotic	Untreated Media		TE-Treated Media	
		All Isolates	*E. coli* Only	All Isolates	*E. coli* Only
		AUC-1	AUC-2	AUC-3	AUC-4
Penicillins	Ampicillin	0.96	0.98	0.96	0.97
β-Lactam/ β-Lactamase Inhib. Combo	Ampicillin–sulbactam	0.89	0.84	0.88	0.85
	Amoxicillin–clavulanic acid	0.88	0.80	0.66	0.58
	Piperacillin–tazobactam	-	-	-	-
1st CEP	Cefazolin	0.92	0.89	0.88	0.85
2nd CEP	Cefotetan	0.75	0.60	0.59	0.54
3rd CEP	Ceftriaxone	0.55	0.55	0.69	0.69
	Ceftazidime	0.65	0.65	0.75	0.73
	Cefotaxime	-	-	0.66	0.69
4th CEP	Cefepime	-	-	0.66	0.69
Carbapenems	Ertapenem	-	-	0.52	0.53
	Meropenem	0.49	0.49	-	-
Monobactams	Aztreonam	-	-	0.66	0.69
Amino- glycosides	Amikacin	-	-	-	-
	Gentamicin	-	-	0.53	0.54
	Tobramycin	0.53	0.55	0.53	0.54
Fluoroquinolones	Ciprofloxacin	0.59	0.55	0.63	0.67
	Levofloxacin	0.58	0.55	0.64	0.68
Folate Pathway Inhibitor	Trimethoprim– sulfamethoxazole	0.53	0.55	0.72	0.73
Tetracycline	Tetracycline	0.69	0.68	0.45	-
Nitrofurans	Nitrofurantoin	0.71	0.59	0.53	-

**Table 9:** The antibiotic categories, agents, and breakpoints used in this study for antibiotic susceptibly testing, following FDA guidelines developed for clinical isolates.

Category	Antibiotic	Abbreviation	Breakpoints ^1^ (µg/mL)		
			S	I	R
Penicillins	Ampicillin	AM	≤8	16	≥32
Penicillins + β-lactam. inhibitor	Ampicillin–sulbactam	A/S	≤8/4	16/8	≥32/16
	Amoxicillin–clavulanic acid	AUG	≤8/4	16/8	≥32/16
AntipseudomonalPenicillins + β-lactam. inhibitor	Piperacillin–tazobactam	P/T	≤16/4	32/4–64/4	≥128/2
Cephalosporins–1st generation	Cefazolin	CFZ	≤8	16	≥32
Cephamycin	Cefotetan	CTN	≤16	32	≥64
Cephalosporins–3rd generation and 4th generation	Ceftriaxone	CAX	<1	2	≥4
	Ceftazidime	CAZ	≤8	16	≥32
	Cefotaxime	CFT	≤8	16–32	≥64
	Cefepime	CPE	≤8	16	≥32
Carbapenems	Ertapenem	ETP	≤2	4	≥8
	Meropenem	MER	≤4	8	≥16
Monobactams	Aztreonam	AZT	≤8	16	≥32
Aminoglycosides	Amikacin	AK	≤16	32	≥64
	Gentamicin	GM	≤4	8	≥16
	Tobramycin	TO	≤4	8	≥16
Fluoroquinolones	Ciprofloxacin	CP	≤1	2	≥4
	Levofloxacin	LVX	≤2	4	≥8
Folate pathway inhibitors	Trimethoprim– sulfamethoxazole	T/S	≤2/38	-	≥4/76
Tetracyclines	Tetracycline	TE	≤4	8	≥16
Nitrofurans	Nitrofurantoin	FD	≤32	64	≥128

^1^ FDA breakpoints for susceptible (S), intermediate (I), and resistant (R) as programmed into MicroScan LabPro V.4.42 for clinical isolates of most Enterobacterales as part of analyses using Negative Urine Combo 85 Panel (Beckman Coulter Catalog No. B1017-435).

## Data Availability

The original data presented in the study are openly available as Supplementary Material via Supplementary File S2.

## Supplementary Materials

The following supporting information can be downloaded at: https://www.mdpi.com/article/10.3390/antibiotics15070709/s1, Figure S1. Antibiotic susceptibility profiles of all isolates (*E. coli* and non-*E. coli*) recovered from untreated and tetracycline-treated media, showing the percentages of isolates classified as susceptible, intermediate, or resistant for 15 antibiotics. Figure S2. Cephalosporin susceptibility profiles of all isolates (*E. coli* and non-*E. coli*) recovered from untreated and tetracycline-treated media, showing the percentages of isolates classified as susceptible, intermediate, or resistant for six cephalosporins. Figure S3. Locations of multidrug-resistant (MDR) isolates recovered in Madison and Estill Counties, Kentucky, and land cover characteristics of Madison County. Figure S4. Locations of multidrug-resistant (MDR) isolates recovered in Madison and Estill Counties, Kentucky, and land cover characteristics of Estill County. Figure S5. Location of a multidrug-resistant (MDR) isolates recovered in Rowan County, Kentucky, and associated land cover characteristics. Figure S6. Geographic distribution of multidrug-resistant (MDR) isolatecollection sites within Kentucky. Figure S7. Topographic map of the watershed associated with an extensively drug-resistant (XDR) *Escherichia coli* isolate recovered in Estill County, Kentucky. Figure S8. Aerial image of the stream location where an extensively drug-resistant (XDR) *Escherichia coli* isolate was recovered. Figure S9. Land cover characteristics of the watershed associated with an extensively drug-resistant (XDR) *Escherichia coli* isolate recovered from White Oak Creek in Estill County, Kentucky. Table S1. Number and percentage of *Escherichia coli* isolates susceptible (S) and non-susceptible (NS) to each antibiotic among all *E. coli* isolates recovered from tetracycline-treated and untreated media. Table S2. Number and percentage of all isolates (including *E. coli* and non-*E. coli*) susceptible (S) and non-susceptible (NS) to each antibiotic among isolates recovered from tetracycline-treated and untreated media. Table S3. Comparison of antibiotic susceptibility among *E. coli* isolates recovered from tetracycline-treated and untreated media, including Holm–Bonferroni-adjusted *p*-values. Table S4. Comparison of antibiotic susceptibility among all isolates (including non-*E. coli*) recovered from tetracycline-treated and untreated media, including Holm–Bonferroni-adjusted *p*-values. Table S5. Distribution of bacterial species recovered from untreated media and their frequency of non-susceptibility across antibiotic classes used to determine multidrug resistance (MDR). Table S6. Distribution of bacterial species recovered from tetracycline-treated media and their frequency of non-susceptibility across antibiotic classes used to determine multidrug resistance (MDR). Table S7. Antibiotic non-susceptibility among *E. coli* isolates stratified by recovery media (untreated versus tetracycline-treated) and microbiological water quality (within or above the quantification range of the ColiGlow test). Table S8. Antibiotic non-susceptibility among all isolates (including non-*E. coli*) stratified by recovery media (untreated versus tetracycline-treated) and microbiological water quality (within or above the quantification range of the ColiGlow test). Table S9. Diagnostic performance of antibiotic non-susceptibility for predicting multidrug resistance among all isolates, including sensitivity, specificity, and area under the receiver operating characteristic (ROC) curve. Table S10. Comparison of antibiotic non-susceptibility prevalence among isolates recovered from untreated ColiGlow, untreated Colilert, and tetracycline-treated ColiGlow media. Table S11. Comparison of *E. coli* species identification by recovery media. Table S12. Distribution of recovered *E. coli* and non-*E. coli* isolates by recovery media according to the number of antibiotic classes exhibiting non-susceptibility for determining multidrug resistance (MDR). Table S13. Distribution of recovered *E. coli* isolates by recovery media according to the number of antibiotic classes exhibiting non-susceptibility for determining multidrug resistance (MDR). Supplementary File S1. MicroScan Panel Alert Report for potential XDR isolate inclusive of the biochemical results and minimum inhibitory concentration (MIC). Supplementary File S2. A spreadsheet file (.xlsx) containing the data for 151 isolates organized by variables (columns) on sheet 1 (CodeSheet).

## Abbreviations

The following abbreviations are used in this manuscript:

AK	Amikacin
AM	Ampicillin
AMO	Amoxicillin
AMR	Antimicrobial resistance
ARGs	Antibiotic resistance genes
AST	Antibiotic susceptibility testing
A/S	Ampicillin–sulbactam
AUC	Area under the ROC curve
AUG	Amoxicillin–clavulanic acid
AZT	Aztreonam
CAZ	Ceftazidime
CAX	Ceftriaxone
CDC	Centers for Disease Control and Prevention
CFT	Cefotaxime
CFU	Colony forming unit
CFZ	Cefazolin
CLSI	Clinical and Laboratory Standards Institute
CP	Ciprofloxacin
CPE	Cefepime
CTN	Cefotetan
EPA	United States Environmental Protection Agency
ESBL	Extended-spectrum β-lactamase
ETP	Ertapenem
EUCAST	European Committee on Antimicrobial Susceptibility Testing
EWG	Environmental Working Group
FDA	Food and Drug Administration
FD	Nitrofurantoin
FSMA	Food Safety Modernization Act
GM	Gentamicin
IMP	Imipenemase gene family
LVX	Levofloxacin
MALDI	Matrix-assisted laser desorption/ionization (time-of-flight mass spectrometry)
MDR	Multidrug resistant/multidrug resistance
MER	Meropenem
MIC	Minimum inhibitory concentration
mTEC	Modified membrane thermotolerant *Escherichia coli* agar
MPN	Most probable number
MRLC	Multi-Resolution Land Characteristics Consortium
MUG	4-methylumbelliferyl-β-D-glucuronide
NARMS	National Antimicrobial Resistance Monitoring System
NS	Non-susceptible/non-susceptibility
ONPG	o-Nitrophenyl-β-D-galactopyranoside
P/T	Piperacillin–tazobactam
PCR	Polymerase chain reaction
ROC	Receiver operating characteristic
RUO	Research use only
S	Susceptible
STEC	Shiga toxin-producing *Escherichia coli*
T/S	Trimethoprim–sulfamethoxazole
TE	Tetracycline
TO	Tobramycin
USDA	United States Department of Agriculture
UV	Ultraviolet
VIM	Verona integron-encoded metallo-β-lactamase
VRE	Vancomycin-resistant *Enterococcus*
WHO	World Health Organization
XDR	Extensively drug resistant
