# Supplementary material for: Occurrence of Phenotypic Multidrug-Resistant E. coli in Kentucky (USA) Surface Waters and Exploration of Sentinel Antibiotics for One Health Surveillance
Source: Antibiotics (Basel). 2026 Jul 21;15(7):709. doi: 10.3390/antibiotics15070709 (PMC13405762; doi:10.3390/antibiotics15070709)
Supplement: Supplementary file 1 [file antibiotics-15-00709-s001.zip › SupplementalFile_XDR_MicroScanReport.pdf]

Panel Alert Report

Patient Name:		Panel Type:	Neg Urine Combo 85
Patient ID:	477_2	Collect Date:	
Specimen/Isolate:	477_2-1	Test Date:	11/27/2024
Source:			
Ward of Isolation:			

Current Alerts

Possible ertapenem-intermediate or resistant Enterobacteriaceae
Unusual resistance. Verify isolate results by repeat testing unless patient had isolate previously.
Save isolate. Infectious Disease consult suggested.

Azt interpretation difference for Enterobacteriaceae: I or R using CLSI BPs 4/8/16
Verify results if warranted.

Caz interpretation difference for Enterobacteriaceae: R using CLSI BPs 4/8/16
Verify results if warranted.

Cft interpretation difference for Enterobacteriaceae: R using CLSI BPs 1/2/4
Verify results if warranted.

Cfz interpretation difference for Enterobacteriaceae: R using CLSI BPs 2/4/8
Verify results if warranted.

Cpe interpretation difference for Enterobacteriaceae: R using CLSI BPs 2/4-8SDD/16
Verify results if warranted.

Possible ESBL – Unable to interpret confirmation test
Possible ESBL. Organism has MICs greater than highest dilution on panel.

Possible ESBL – Unable to interpret confirmation test
Possible ESBL. Organism has MICs greater than highest dilution on panel.

WGS

repeated

possible ESBL

Panel Data

Biotype: 73015012174

Organism Identification:

	Organism	% Probability	Footnotes	Special Characteristics
1	E. coli	99.99		

Biochemical Results: (Biochemicals that are bolded and underlined are atypical for the first choice organism)

GLU	+	RAF	-	INO	-	URE	-	LYS	+	TDA	-	CIT	-	CL4	-	TAR	-	OF/G	+	NIT	+
SUC	+	RHA	+	ADO	-	H2S	-	ARG	-	ESC	-	MAL	-	CF8	+	ACE	-	P4	+	FD64	-
SOR	+	ARA	+	MEL	-	IND	+	ORN	+	VP	-	ONPG	+	TO4	-	**CET**	+	K4	+	OXI	-

MIC Results: (Antimicrobics marked with "Ø" are suppressed from Long and Short Format Patient Reports)

A/S	AK	AM	AUG	AZT	CAX	CAZ	ØCAZ/CA	CFT	ØCFT/CA	ØCFTE	CFZ	CP	CPE	CTN
>16/8	<=16	>16	>16/8	>16	>32	>16	>2	>32	>4	>1	>16	>2	>16	32
R	S	R	R	R	R	R		R			R	R	R	I

ETP	FD	GM	LVX	MER	ØMIN	P/T	T/S	TE	TO
1	<=32	<=4	>4	<=1	8	<=16	>2/38	>8	<=4
I		S	R	S	I	S*	R	R	S

Extra Tests: ESBL -

Alert History

Possible ertapenem-intermediate or resistant Enterobacteriaceae
"Unusual resistance","Verify isolate results by repeat testing unless patient had isolate previously","Save isolate","Infectious Disease consult suggested"

Alert Resolution:

Azt interpretation difference for Enterobacteriaceae: I or R using CLSI BPs 4/8/16
"Verify results if warranted"

Alert Resolution:

Caz interpretation difference for Enterobacteriaceae: R using CLSI BPs 4/8/16
"Verify results if warranted"

Alert Resolution:

^ - Reported Interpretation Changed
