# Supplementary material for: Occurrence of Phenotypic Multidrug-Resistant E. coli in Kentucky (USA) Surface Waters and Exploration of Sentinel Antibiotics for One Health Surveillance
Source: Antibiotics (Basel). 2026 Jul 21;15(7):709. doi: 10.3390/antibiotics15070709 (PMC13405762; doi:10.3390/antibiotics15070709)
Supplement: Supplementary file 1 [file antibiotics-15-00709-s001.zip › Supplementary Figures July 16.pdf]

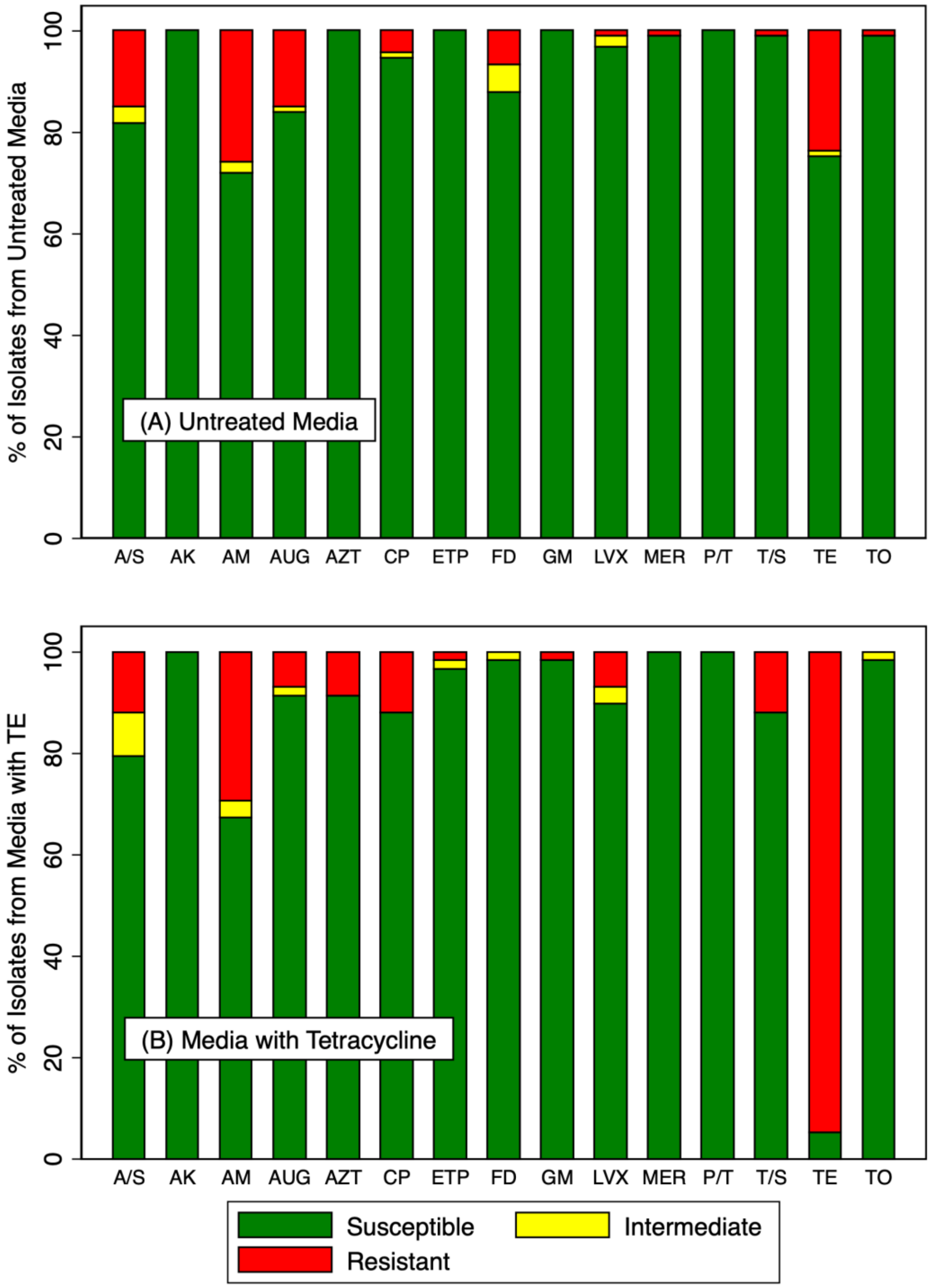

Figure S1. Antibiotic susceptibility profiles presented as stacked barcharts illustrating the percentage of all isolates (*E. coli* and non-*E. coli*) exhibiting susceptibility, intermediate resistance, and resistance to 15 antibiotics. (A) Antibiotic susceptibility profile for isolates obtained from media without tetracycline (n ~ 93); (B) antibiotic susceptibility profile for isolates obtained from tetracycline-treated media (n = 58). A/S: ampicillin–sulbactam, AK: amikacin, AM: ampicillin, AUG: amoxicillin–clavulanic acid, AZT: aztreonam, CP: ciprofloxacin, ETP: ertapenem, FD: nitrofurantoin, GM: gentamicin, LVX: levofloxacin, MER: meropenem, P/T: piperacillin–tazobactam, T/S: trimethoprim–sulfamethoxazole, TE: tetracycline, TO: tobramycin.

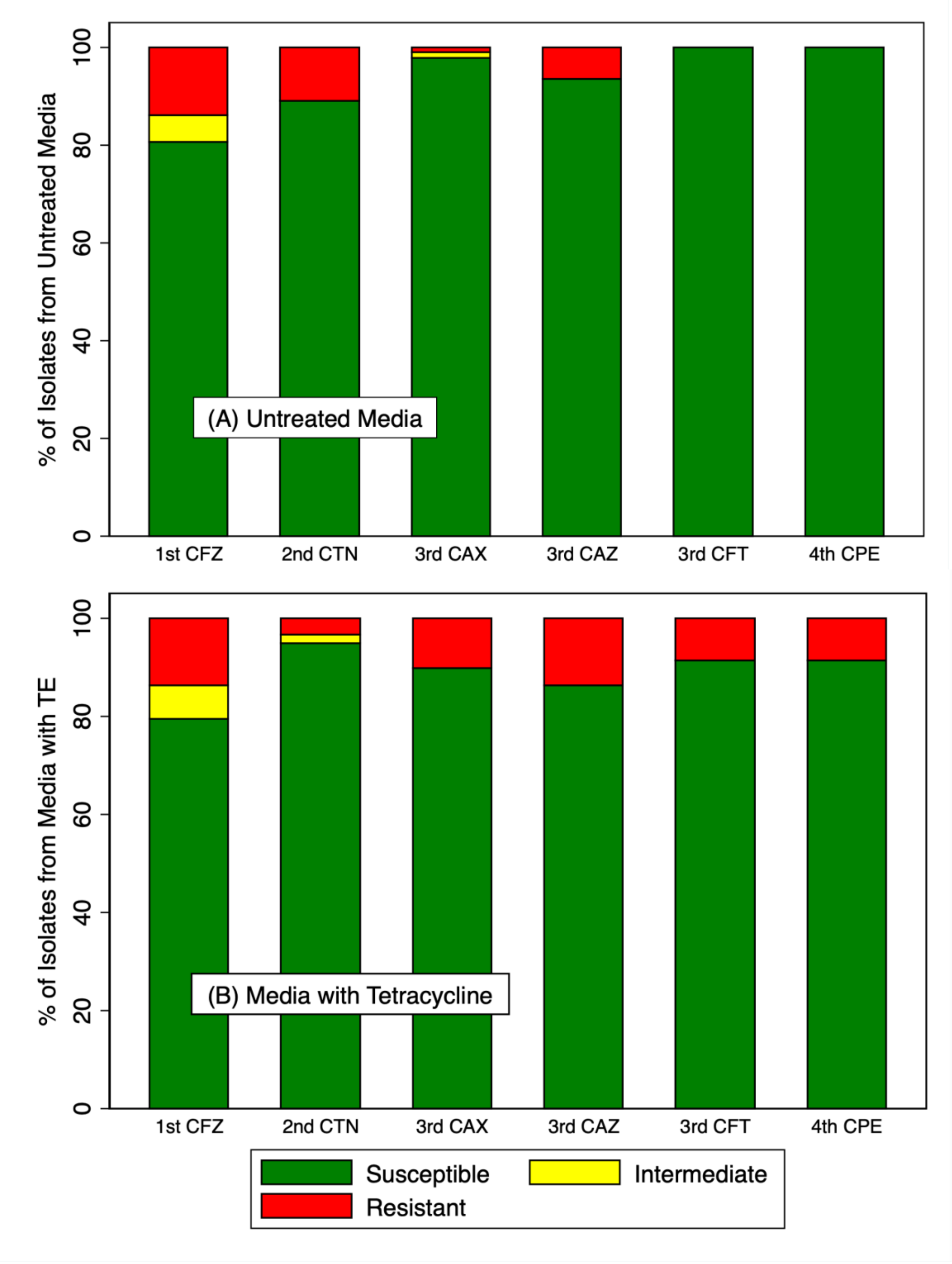

Figure S2. Cephalosporin-specific antibiotic susceptibility profiles for all isolates evaluated illustrating the percentage of all isolates (*E. coli* and non-*E.*coli) exhibiting susceptibility, intermediate resistance, and resistance to six antibiotics. (A) Cephalosporin susceptibility profile for isolates obtained from media without tetracycline (*n* ~ 93); (B) cephalosporin susceptibility profile for isolates obtained from tetracycline-treated media (*n* = 58) CFZ: cefazolin, CTN: cefotetan, CAX: ceftriaxone, CAZ: ceftazidime, CFT: cefotaxime, CPE: cefepime

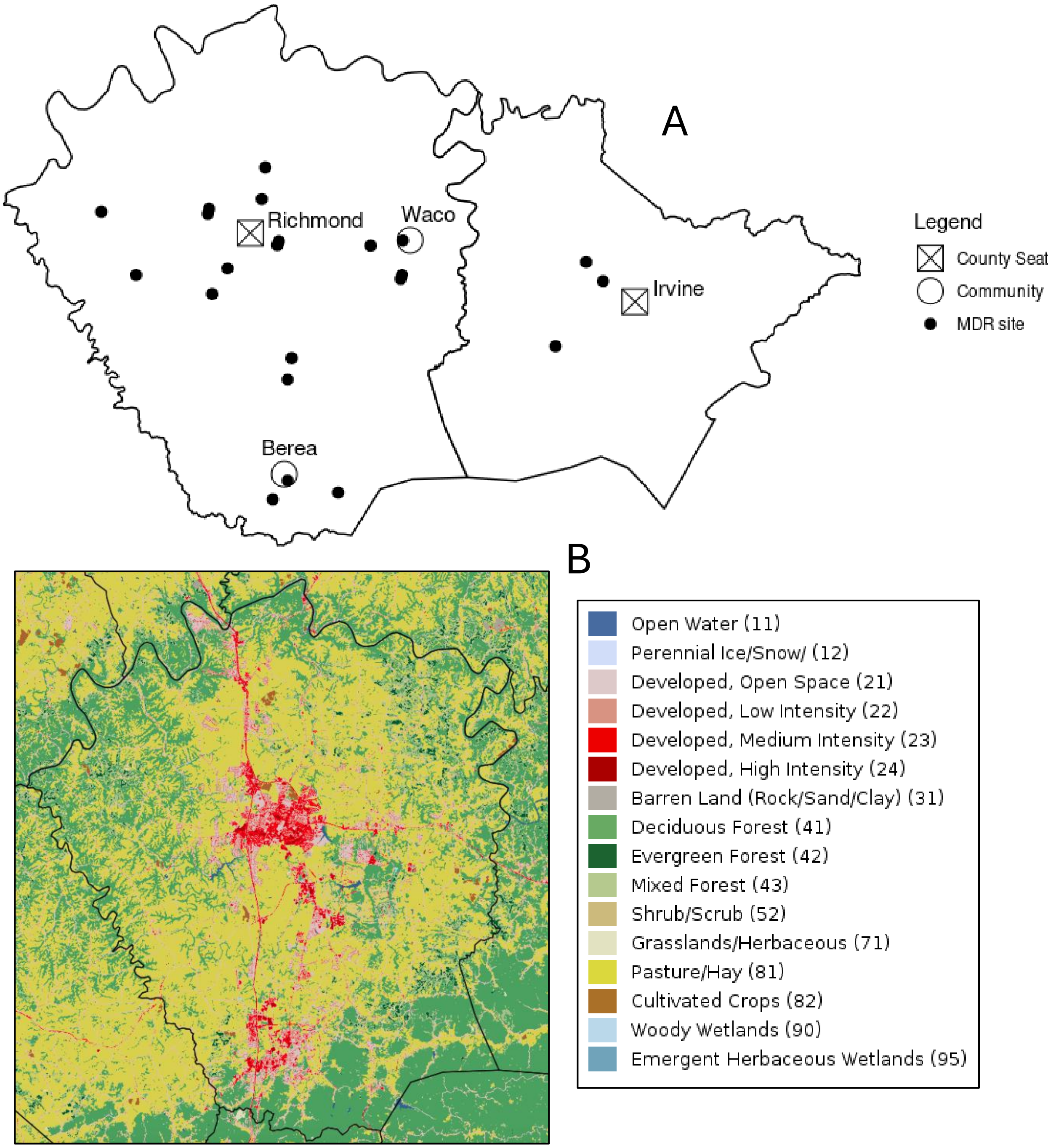

Figure S3. Maps illustrating location of where multidrug resistant (MDR) isolates were obtained within two Kentucky counties. (**A**) MDR isolates obtained in Madison County (left/West) and Estill County (right/East) were recovered from small streams proximal to the black dots. (**B**) Madison County Land Cover map presenting 30-meter spatial resolution of 2024 land cover with the 16 Anderson Level II classes obtained from the US Multi-Resolution Land Characteristics (MRLC) Consortium via the National Land Cover Database viewer.

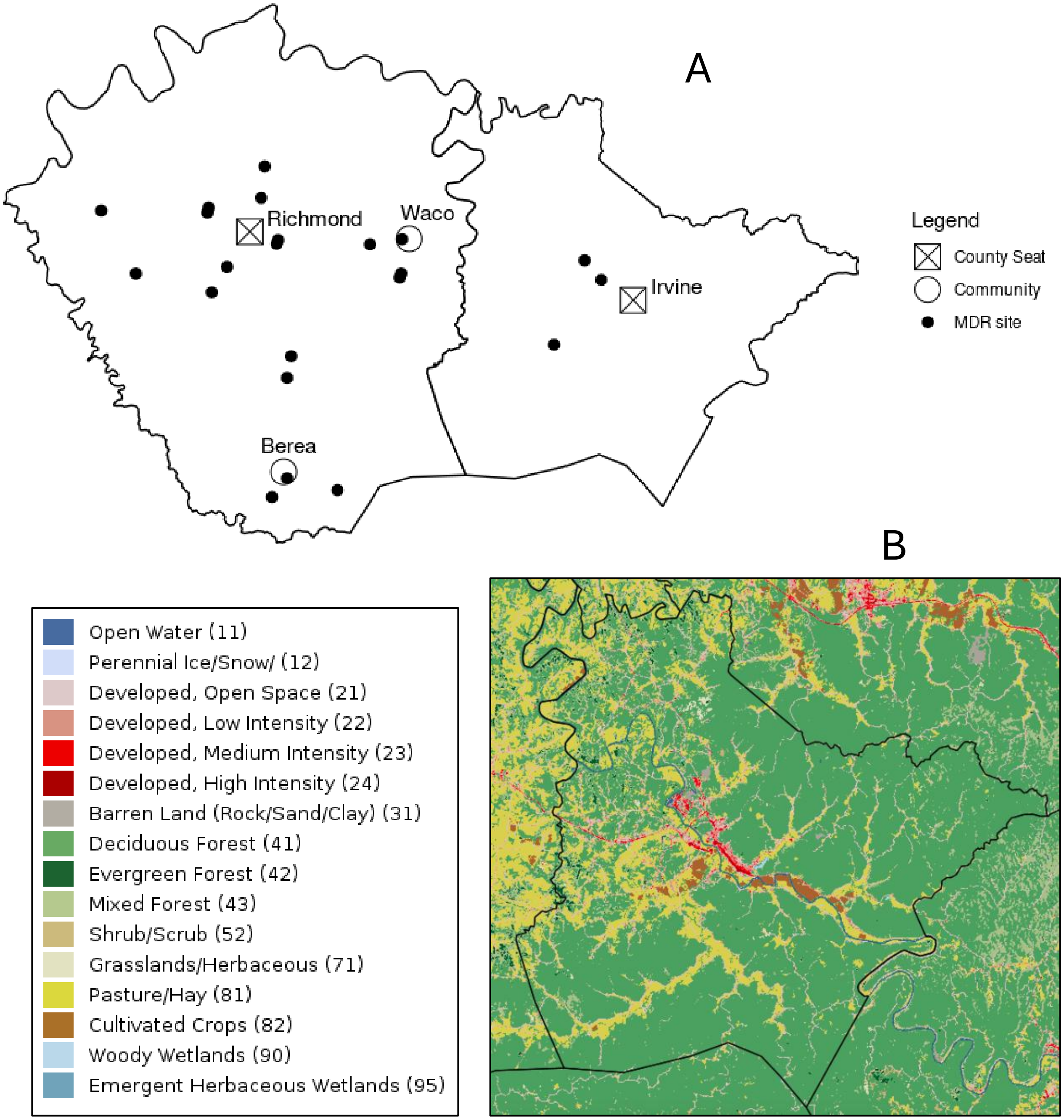

Figure S4. Maps illustrating location of where multidrug resistant (MDR) isolates were obtained within two Kentucky counties. (**A**) MDR isolates obtained in Madison County (left/West) and Estill County (right/East) were collected from streams proximal to the black dots. (**B**) Estill County Land Cover map presenting 30-meter spatial resolution of 2024 land cover with the 16 Anderson Level II classes obtained from the US Multi-Resolution Land Characteristics (MRLC) Consortium via the National Land Cover Database viewer.

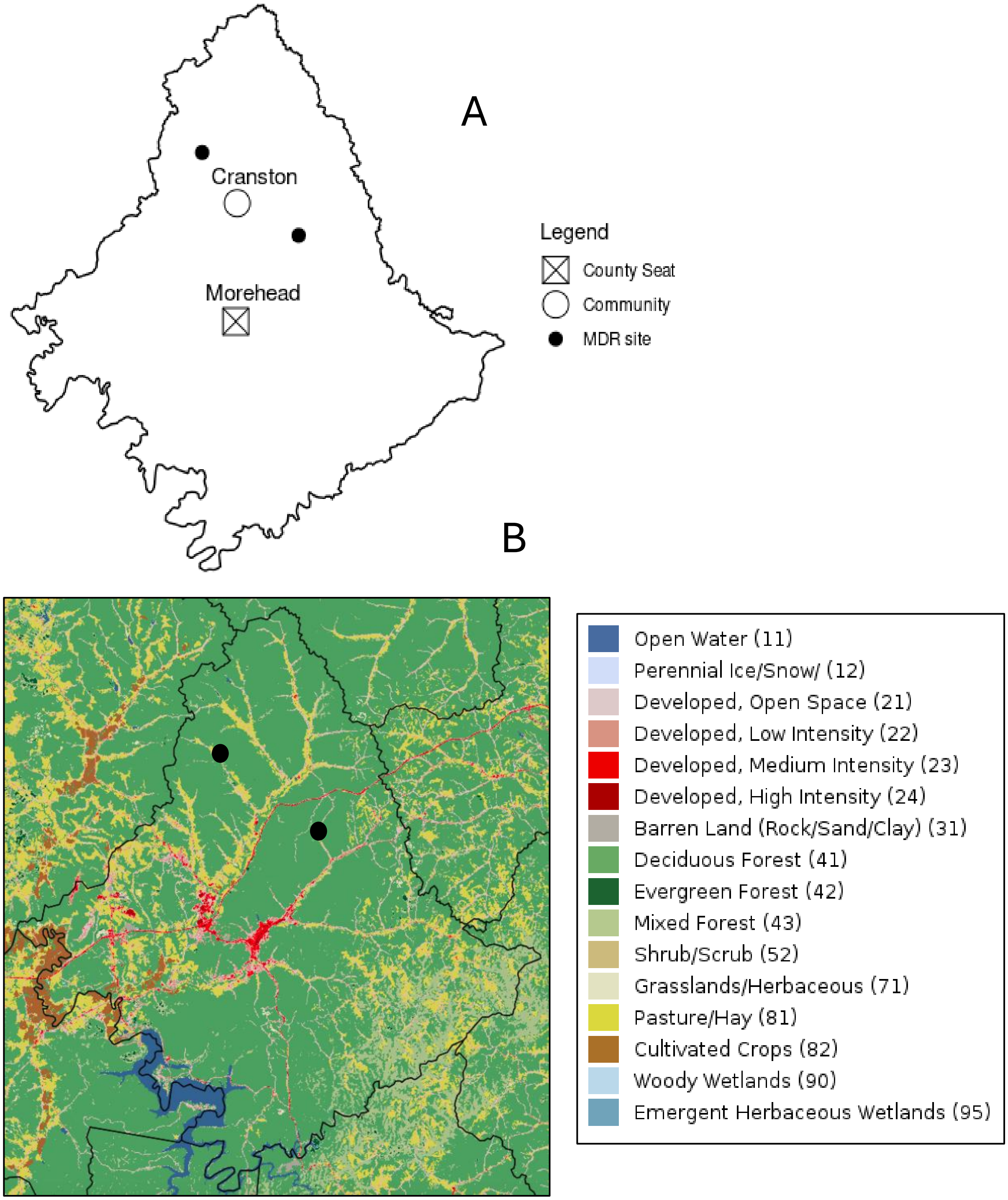

Figure S5. Maps of location of one multidrug resistant (MDR) isolate in Rowan County, Kentucky. (**A**) The MDR isolate was obtained from a small stream proximal to the black dot. (**B**) Rowan County Land Cover map presenting 30-meter spatial resolution of 2024 land cover with the 16 Anderson Level II classes obtained from the US Multi-Resolution Land Characteristics (MRLC) Consortium via the National Land Cover Database viewer.

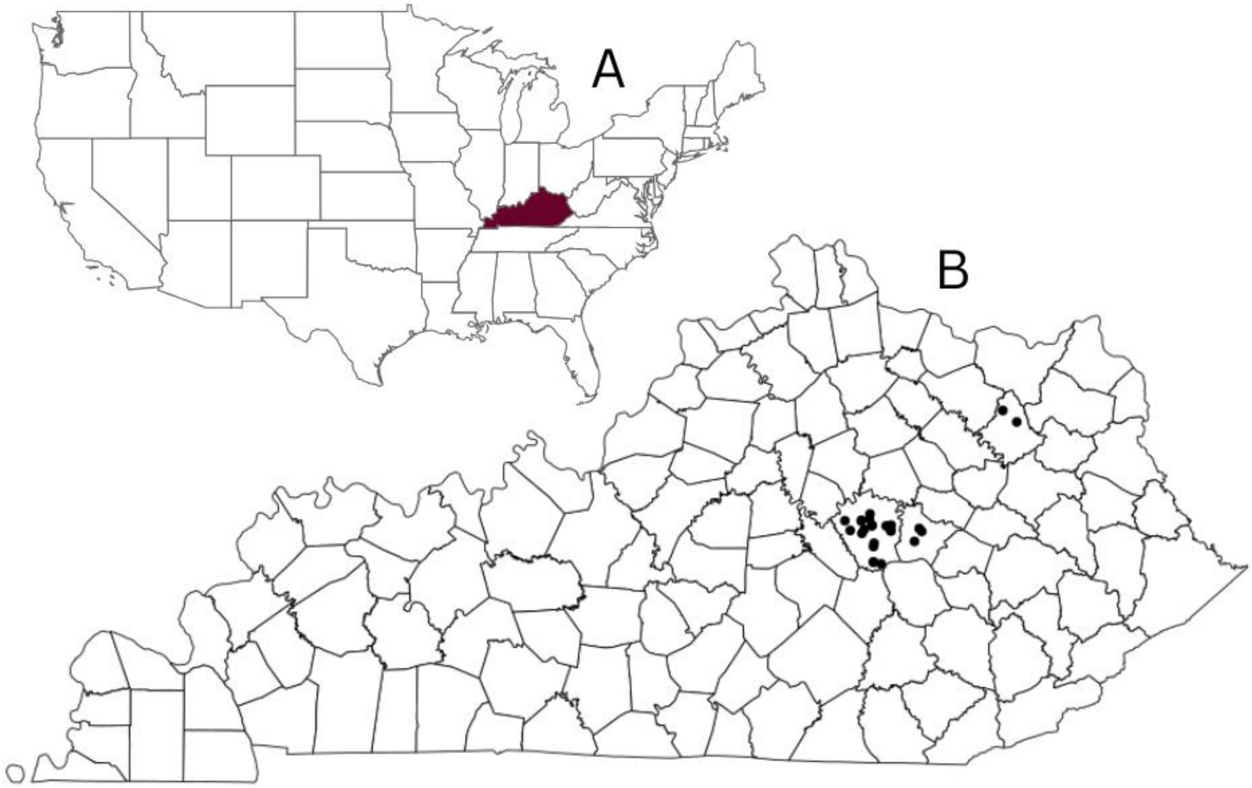

Figure S6. Map illustrating location of where multidrug resistant (MDR) isolates were obtained. (A) Samples in this study were collected in the Commonwealth of Kentucky, which is the maroon-colored US state. (B) The MDR isolate locations are depicted with black dots which are inside Madison, Estill, and Rowan Counties in Kentucky.

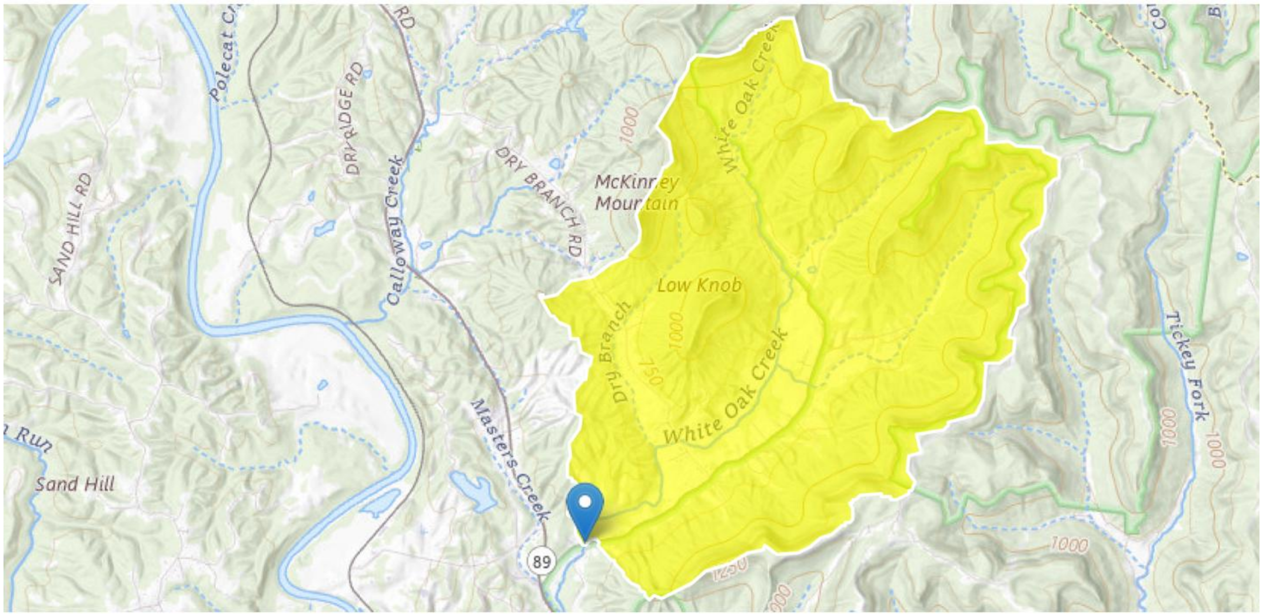

Figure S7. Topographic map (with contour labels in feet above sea level) related to one extensively drug resistant (XDR) *E. coli* isolate recovered in Estill County, Kentucky, with drainage area (catchment) above the sample collection point highlighted in yellow.

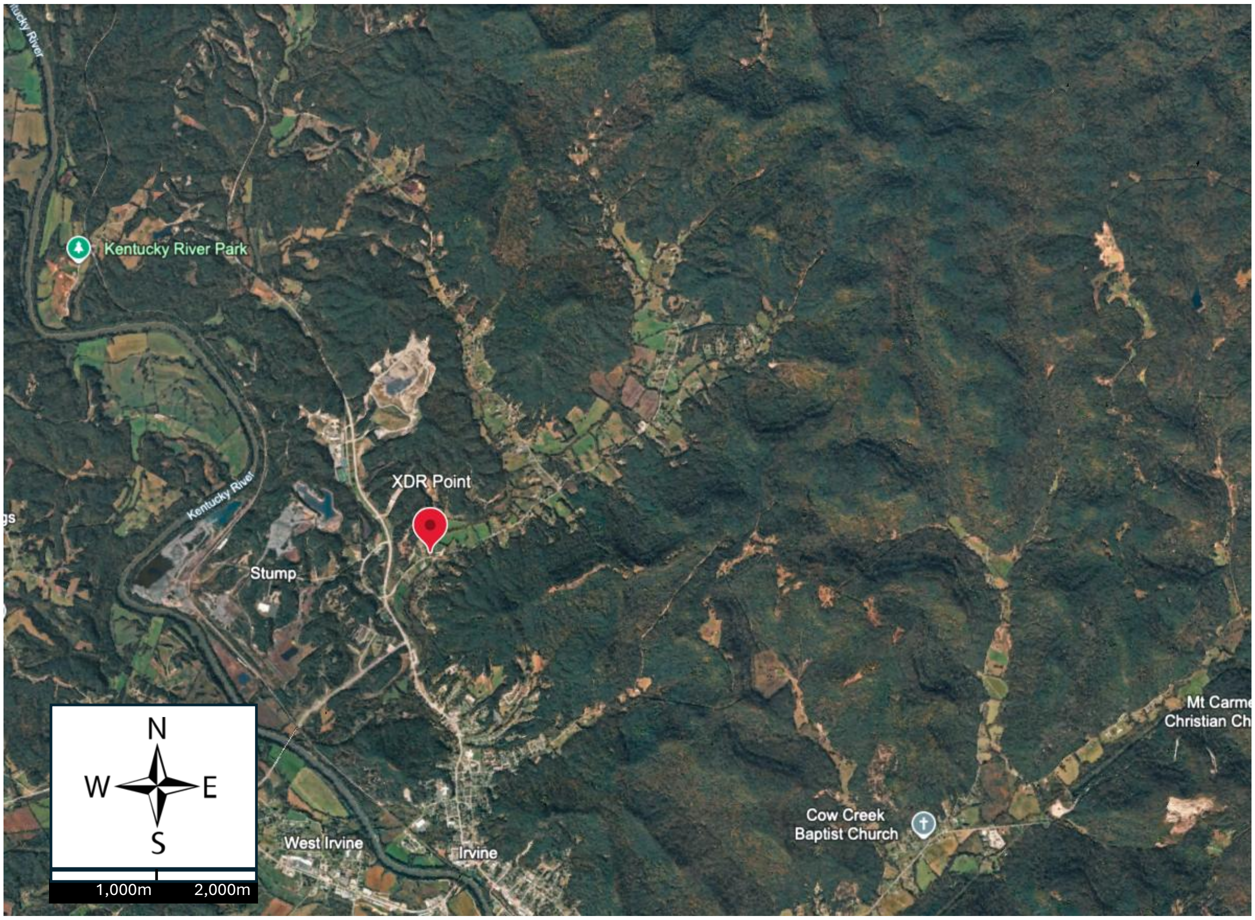

Figure S8. Aerial image (Google Earth) illustrating location of stream whereby extensively drug resistant (XDR) *E. coli* isolate was recovered.

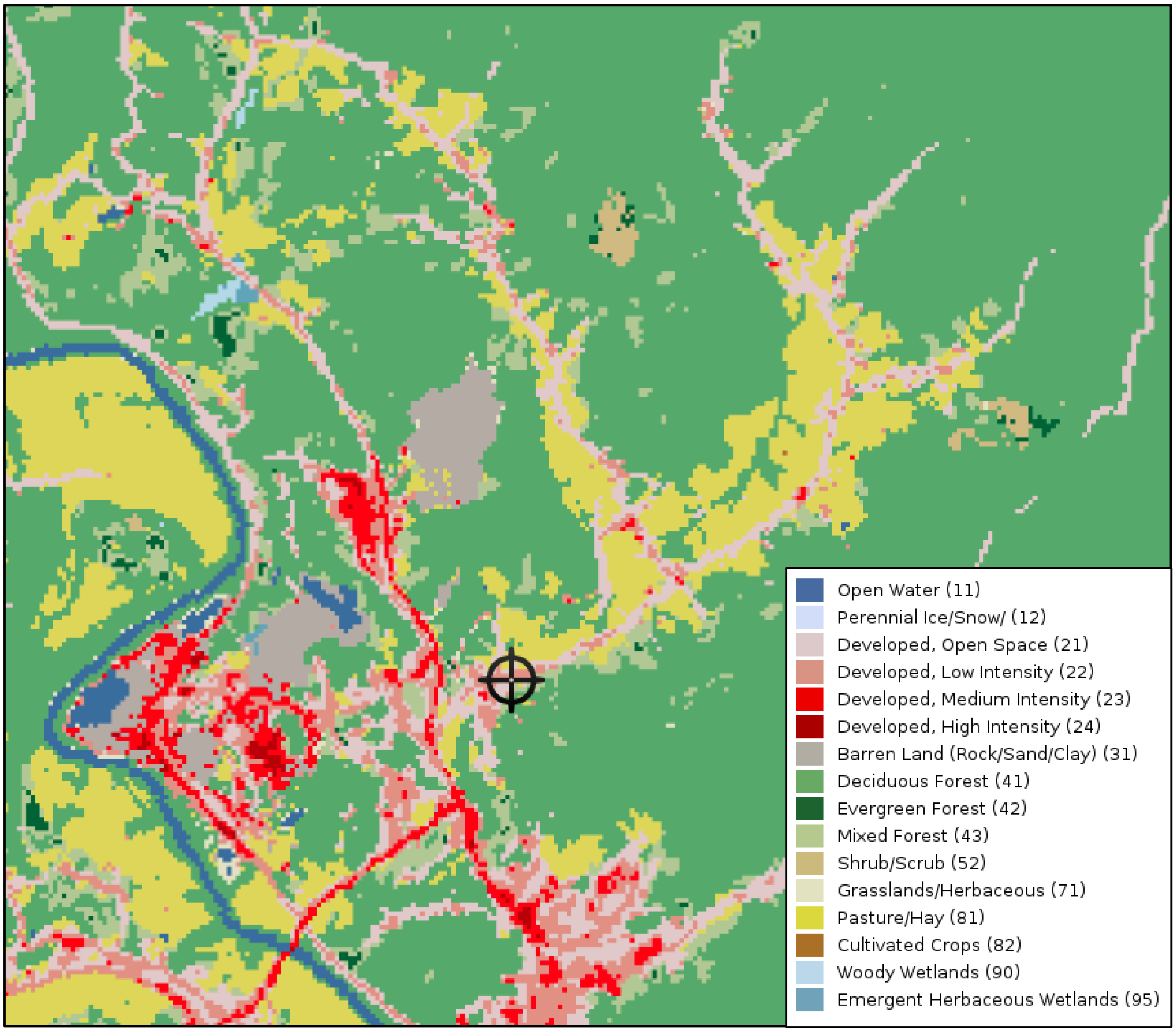

Figure S9. Land cover map related to one extensively drug resistant (XDR) *E. coli* isolate recovered in Estill County, Kentucky; whereby the XDR isolate was obtained from White Oak Creek proximal to the black crosshair. The land cover map is presenting 30-meter spatial resolution of 2024 land cover with the 16 Anderson Level II classes obtained from the US Multi-Resolution Land Characteristics (MRLC) Consortium via the National Land Cover Database viewer.
