# Supplementary material for: Occurrence of Phenotypic Multidrug-Resistant E. coli in Kentucky (USA) Surface Waters and Exploration of Sentinel Antibiotics for One Health Surveillance
Source: Antibiotics (Basel). 2026 Jul 21;15(7):709. doi: 10.3390/antibiotics15070709 (PMC13405762; doi:10.3390/antibiotics15070709)
Supplement: Supplementary file 1 [file antibiotics-15-00709-s001.zip › Supplementary Tables Revision.pdf]

Table S1. The number and percent of isolates determined to be susceptible (S) or non-susceptible (NS) to individual antibiotics inclusive of all 128 *E. coli* isolates obtained from tetracycline-treated (*n* = 52) and untreated media (*n* = 75).

Antibiotic	Abbrev.	All *E. coli* Isolates				
		n	**S**	**% S**	**NS**	**% NS**
Ampicillin	AM	128	101	79.53	26	20.47
Ampicillin–sulbactam	A/S	128	110	86.61	17	13.39
Amoxicillin–clavulanic acid	AUG	128	119	93.70	8	6.30
Piperacillin–tazobactam	P/T	128	127	100.00	0	0.00
Cefazolin	CFZ	128	109	85.83	18	14.17
Cefotetan	CTN	128	124	97.64	3	2.36
Ceftriaxone	CAX	128	121	95.28	6	4.72
Ceftazidime	CAZ	128	118	92.91	9	7.09
Cefotaxime	CFT	128	122	96.06	5	3.94
Cefepime	CPE	128	122	96.06	5	3.94
Ertapenem	ETP	128	125	98.43	2	1.57
Meropenem	MER	128	126	99.21	1	0.79
Aztreonam	AZT	128	122	96.06	5	3.94
Amikacin	AK	128	127	100.00	0	0.00
Gentamicin	GM	128	126	99.21	1	0.79
Tobramycin	TO	128	125	98.43	2	1.57
Ciprofloxacin	CP	128	120	94.49	7	5.51
Levofloxacin	LVX	128	121	95.28	6	4.72
Trimethoprim–sulfamethoxazole	T/S	128	121	95.28	6	4.72
Tetracycline	TE	128	61	48.03	66	51.97
Nitrofurantoin	FD	128	124	97.64	3	2.36

Table S2. The number and percent of isolates determined to be susceptible (S) or non-susceptible (NS) to individual antibiotics inclusive of all 151 isolates (including *E. coli* and non-*E. coli*) obtained from tetracycline-treated (n = 58) and untreated media (n ~ 93).

**Antibiotic**	**Abbrev.**	**All Isolates including non-*E. coli* Isolates**				
		N	**S**	**% S**	**NS**	**% NS**
Ampicillin	AM	151	106	70.20	45	29.80
Ampicillin–sulbactam	A/S	151	122	80.79	29	19.21
Amoxicillin–clavulanic acid	AUG	151	131	86.75	20	13.25
Piperacillin–tazobactam	P/T	151	151	100.00	0	0.00
Cefazolin	CFZ	151	121	80.13	30	19.87
Cefotetan	CTN	149	136	91.28	13	8.72
Ceftriaxone	CAX	151	143	94.70	8	5.30
Ceftazidime	CAZ	151	137	90.73	14	9.27
Cefotaxime	CFT	151	146	96.69	5	3.31
Cefepime	CPE	151	146	96.69	5	3.31
Ertapenem	ETP	151	149	98.68	2	1.32
Meropenem	MER	151	150	99.34	1	0.66
Aztreonam	AZT	151	146	96.69	5	3.31
Amikacin	AK	149	149	100.00	0	0.00
Gentamicin	GM	149	148	99.33	1	0.67
Tobramycin	TO	149	147	98.66	2	1.34
Ciprofloxacin	CP	151	139	92.05	12	7.95
Levofloxacin	LVX	151	142	94.04	9	5.96
Trimethoprim–sulfamethoxazole	T/S	151	143	94.70	8	5.30
Tetracycline	TE	151	78	51.66	73	48.34
Nitrofurantoin	FD	149	137	91.95	12	8.05

Table S3. Focusing only on isolates identified as *E. coli,* comparison of the number and percent of isolates susceptible (S) or non-susceptible (NS) to individual antibiotics in tetracycline-treated (*n* = 53) and untreated media (*n* ~ 77), accompanied with a *p*-value assessing a difference in the frequency of non-susceptibility between the media types.

**ABX**	**Tetracycline-Treated Media Isolates**						**Untreated Media Isolates**					**P[1]**	**Holm-adj. *P***
	n	**S**	**% S**	**NS**	**% NS**		***n***	**S**	**% S**	**NS**	**% NS**		
AM	53	38	71.7	15	28.3		77	65	84.4	12	15.6	0.079	0.869
A/S	53	44	83.0	9	17.0		77	69	89.6	8	10.4	0.273	1.000
AUG	53	51	96.2	2	3.8		77	71	92.2	6	7.8	0.471	1.000
P/T	53	53	100.0	0	0.0		77	77	100.0	0	0.0	—	—
CFZ	53	44	83.0	9	17.0		77	68	88.3	9	11.7	0.391	1.000
CTN	53	52	98.1	1	1.9		75	73	97.3	2	2.7	1.000	1.000
CAX	53	48	90.6	5	9.4		77	76	98.7	1	1.3	0.041	0.492
CAZ	53	47	88.7	6	11.3		77	74	96.1	3	3.9	0.158	1.000
CFT	53	48	90.6	5	9.4		77	77	100.0	0	0.0	0.010	0.170
CPE	53	48	90.6	5	9.4		77	77	100.0	0	0.0	0.010	0.170
ETP	53	51	96.2	2	3.8		77	77	100.0	0	0.0	0.164	1.000
MER	53	53	100.0	0	0.0		77	76	98.7	1	1.3	1.000	1.000
AZT	53	48	90.6	5	9.4		77	77	100.0	0	0.0	0.010	0.170
AK	53	53	100.0	0	0.0		75	75	100.0	0	0.0	—	—
GM	53	52	98.1	1	1.9		75	75	100.0	0	0.0	0.414	1.000
TO	53	52	98.1	1	1.9		75	74	98.7	1	1.3	1.000	1.000
CP	53	46	86.8	7	13.2		77	76	98.7	1	1.3	0.008	0.144
LVX	53	47	88.7	6	11.3		77	76	98.7	1	1.3	0.018	0.252
T/S	53	47	88.7	6	11.3		77	76	98.7	1	1.3	0.018	0.252
TE	53	0	0.0	53	100.0		77	63	81.8	14	18.2	<0.001	***< 0.019***
FD	53	53	100.0	0	0.0		75	72	96.0	3	4.0	0.266	1.000

[1]*p* Represents the *p*-value obtained from the Chi-Square Test or the Fisher's Exact Test; whereby the Chi-Square Test was used if all 2 x 2 table cell counts $\geq$ 5 and the Exact Test is used when any cell count < 5.

Table S4. Using all isolates (including non-*E. coli*), comparison of the number and percent of isolates susceptible (S) or non-susceptible (NS) to individual antibiotics in tetracycline-treated (n = 58) and untreated media (n ~ 93), accompanied with a p-value assessing a difference in the frequency of non-susceptibility between the media types.

**ABX**	**Tetracycline-Treated Media Isolates**						**Untreated Media Isolates**					***P*[1]**	**Holm-adj. *P***
	n	**S**	**% S**	**NS**	**% NS**		***n***	**S**	**% S**	**NS**	**% NS**		
AM	58	39	67.24	19	32.76		93	67	72.04	26	27.96	0.530	1.000
A/S	58	46	79.31	12	20.69		93	76	81.72	17	18.28	0.715	1.000
AUG	58	53	91.38	5	8.62		93	78	83.87	15	16.13	0.186	1.000
P/T	58	58	100.00	0	0.00		93	93	100.00	0	0.00	N/A	—
CFZ	58	46	79.31	12	20.69		93	75	80.65	18	19.35	0.842	1.000
CTN	58	55	94.83	3	5.17		91	81	89.01	10	10.99	0.178	1.000
CAX	58	52	89.66	6	10.34		93	91	97.85	2	2.15	***0.037***	0.481
CAZ	58	50	86.21	8	13.79		93	87	93.55	6	6.45	0.130	1.000
CFT	58	53	91.38	5	8.62		93	93	100.00	0	0.00	***0.007***	0.119
CPE	58	53	91.38	5	8.62		93	93	100.00	0	0.00	***0.007***	0.119
ETP	58	56	96.55	2	3.45		93	93	100.00	0	0.00	0.146	1.000
MER	58	58	100.00	0	0.00		93	92	98.92	1	1.08	0.616	1.000
AZT	58	53	91.38	5	8.62		93	93	100.00	0	0.00	***0.007***	0.119
AK	58	58	100.00	0	0.00		91	91	100.00	0	0.00	N/A	—
GM	58	57	98.28	1	1.72		91	91	100.00	0	0.00	0.389	1.000
TO	58	57	98.28	1	1.72		91	90	98.90	1	1.10	0.629	1.000
CP	58	51	87.93	7	12.07		93	88	94.62	5	5.38	0.122	1.000
LVX	58	52	89.66	6	10.34		93	90	96.77	3	3.23	0.076	0.912
T/S	58	51	87.93	7	12.07		93	92	98.92	1	1.08	***0.005***	0.090
TE	58	3	5.17	55	94.83		93	70	75.27	23	24.73	***<0.001***	0.019
FD	58	57	98.28	1	1.72		91	80	87.91	11	12.09	***0.019***	0.266

[1]*p* Represents the *p*-value obtained from the Chi-Square Test or the Fisher's Exact Test; whereby the Chi-Square Test was used if all 2 x 2 table cell counts $\geq$ 5 and the Exact Test is used when any cell count < 5.
[2]Antibiotic susceptibility is not determined for all antibiotic classes for *S. enterica* if identified by MicroScan as *Salmonella enterica*; therefore MDR determination was not able to be assessed.

Table S5. Bacterial species observed among 91 isolates recovered from untreated media and their frequency of non-susceptibility (NS) to multiple antibiotic categories for determining multidrug resistance (MDR); whereby MDR is defined as NS to at least one antibiotic in three or more antibiotic categories. *None from untreated media exceeded 6.

**Species**	**No.**	**Antibiotic Categories with Non-Susceptibility**							**No. %MDR**
		**0**	**1**	**2**	**3**	**4**	**5**	**6**	
Citrobacter spp.	1	-	-	-	-	-	1	-	1 (100.00)
E. coli	75	51	14	0	2	7	1	-	10 (13.33)
Enterobacter spp.	7	-	-	-	1	2	2	2	7 (100.00)
Klebsiella spp.	5	1	2	2	-	-	-	-	0 (0.00)
Kluyvera sp.	1	-	-	1	-	-	-	-	0 (0.00)
Serratia sp.	2	-	-	-	-	1	-	1	2 (100.00)
Total	91	52	16	3	3	10	4	3	20 (21.98)

Table S6. Bacterial species observed among 58 isolates recovered from media treated with tetracycline and their frequency of non-susceptibility (NS) to multiple antibiotic categories for determining multidrug resistance (MDR); whereby MDR is defined as NS to at least one antibiotic in three or more antibiotic classes. *None exceeded 10 categories, all were resistant to one, and none were observed for nine.

**Species**	**No.**	**No. of Antibiotic Categories with Non-Susceptibility**										**No. (%MDR)**
		**0**	**1**	**2**	**3**	**4**	**5**	**6**	**7**	**8**	**10**	
E. coli	53	-	35	5	3	5	-	-	1	3	1	13 (24.53)
Enterobacter sp	2	-	-	-	-	-	2	-	-	-	-	2 (100.00)
Klebsiella sp.	3	-	2	-	-	-	1	-	-	-	-	1 (33.33)
Total	149	-	37	5	3	5	3	-	1	3	1	16 (27.59)

Table S7. Antibiotic non-susceptibility among *E. coli* only isolates (n ~ 130) stratified by their origin media (untreated versus tetracycline-treated) and the origin water quality classified as *E. coli* levels being above or within the range of quantification of ColiGlow test method.

Antibiotic	Untreated Media (n ~ 77)			Tetracycline-Treated Media (n = 53)		
	***E. coli* Overrange**[1]	***E. coli* In Range**		***E. coli* Overrange**	***E. coli* In Range**	
	**NS[2]/n (%)**	**NS/n (%)**	p^3	**NS/n (%)**	**NS/n (%)**	p^3
Ampicillin	2/19 (10.5%)	10/58 (17.2%)	0.719	8/30 (26.7%)	7/23 (30.4%)	0.763
Ampicillin–sulbactam	2/19 (10.5%)	6/58 (10.3%)	1.000	5/30 (16.7%)	4/23 (17.4%)	1.000
Amoxicillin–clavulanic acid	1/19 (5.3%)	5/58 (8.6%)	1.000	0/30 (0.0%)	2/23 (8.7%)	0.184
Piperacillin–tazobactam	0/19 (0.0%)	0/19 (0.0%)	-	0/19 (0.0%)	0/23 (0.0%)	-
Cefazolin	1/19 (5.3%)	8/58 (13.8%)	0.438	5/30 (16.7%)	4/23 (17.4%)	1.000
Cefotetan	0/19 (0.0%)	2/56 (3.6%)	1.000	0/30 (0.0%)	1/23 (4.3%)	0.434
Ceftriaxone	0/19 (0.0%)	1/58 (1.7%)	1.000	3/30 (10.0%)	2/23 (8.7%)	1.000
Ceftazidime	1/19 (5.3%)	2/58 (3.4%)	1.000	4/30 (13.3%)	2/23 (8.7%)	0.687
Cefotaxime	0/19 (0.0%)	0/58 (0.0%)	-	3/30 (10.0%)	2/23 (8.7%)	1.000
Cefepime	0/19 (0.0%)	0/58 (0.0%)	-	3/30 (10.0%)	2/23 (8.7%)	1.000
Ertapenem	0/19 (0.0%)	0/58 (0.0%)	-	0/30 (0.0%)	2/23 (8.7%)	0.184
Meropenem	0/19 (0.0%)	1/58 (1.7%)	1.000	0/30 (0.0%)	0/23 (0.0%)	-
Aztreonam	0/19 (0.0%)	0/58 (0.0%)	-	3/30 (10.0%)	2/23 (8.7%)	1.000
Amikacin	0/19 (0.0%)	0/56 (0.0%)	-	0/28 (0.0%)	0/23 (0.0%)	-
Gentamicin	0/19 (0.0%)	0/56 (0.0%)	-	0/30 (0.0%)	1/23 (4.3%)	0.434
Tobramycin	0/19 (0.0%)	1/56 (1.8%)	1.000	0/30 (0.0%)	1/23 (4.3%)	0.434
Ciprofloxacin	0/19 (0.0%)	1/58 (1.7%)	1.000	3/30 (10.0%)	4/23 (17.4%)	0.451
Levofloxacin	0/19 (0.0%)	1/58 (1.7%)	1.000	3/30 (10.0%)	3/23 (13.0%)	1.000
Trimethoprim–sulfamethoxazole	0/19 (0.0%)	1/58 (1.7%)	1.000	3/30 (10.0%)	3/23 (13.0%)	1.000
Tetracycline	2/19 (10.5%)	12/58 (20.7%)	0.497	30/30 (100.0%)	23/23 (100.0%)	-
Nitrofurantoin	2/19 (10.5%)	1/56 (1.8%)	0.156	0/30 (0.0%)	0/23 (0.0%)	-

[1]Overange: *E. coli* density exceeds 1,479 MPN per 100 mL

[2]NS: Not susceptible to antibiotic

[3] *p: p*-value from Fisher Exact tests (if any cell count is ≤ 5) or Chi-Square tests and is not adjusted for multiple comparisons as no significant results exist with unadjusted values.

Table S8. Antibiotic non-susceptibility among all (N = 151) isolates (including non-*E. coli*) stratified by their isolate origin media (untreated versus tetracycline-treated) and the microbiological water quality classified as *E. coli* levels being above or within the range of ColiGlow enumeration test method.

Antibiotic	Untreated Media (n = 93)			Tetracycline-Treated Media (n = 58)		
	E. coli Overrange[1]	*E. coli* In Range		*E. coli* Overrange	*E. coli* In Range	
	NS[2]/n (%)	NS/n (%)	p[3]	NS/n (%)	NS/n (%)	p[3]
Ampicillin	12/29 (41.4%)	14/64 (21.9%)	0.052	10/33 (30.3%)	9/25 (36.0%)	0.647
Ampicillin–sulbactam	8/29 (27.6%)	9/64 (14.1%)	0.118	7/33 (21.2%)	5/25 (20.0%)	0.91
Amoxicillin–clavulanic acid	7/29 (24.1%)	8/64 (12.5%)	0.157	2/33 (6.1%)	3/25 (12.0%)	0.425
Piperacillin–tazobactam	0/29 (0.0%)	0/64 (0.0%)	-	0/33 (0.0%)	0/25 (0.0%)	-
Cefazolin	7/29 (24.1%)	11/64 (17.2%)	0.572	7/33 (21.2%)	5/25 (20.0%)	1.000
Cefotetan	6/29 (20.7%)	4/62 (6.5%)	0.069	1/33 (3.0%)	2/25 (8.0%)	0.572
Ceftriaxone	1/29 (3.4%)	1/64 (1.6%)	0.529	3/33 (9.1%)	3/25 (12.0%)	1.000
Ceftazidime	3/29 (10.3%)	3/64 (4.7%)	0.371	5/33 (15.2%)	3/25 (12.0%)	1.000
Cefotaxime	0/29 (0.0%)	0/64 (0.0%)	-	3/33 (9.1%)	2/25 (8.0%)	1.000
Cefepime	0/29 (0.0%)	0/64 (0.0%)	-	3/33 (9.1%)	2/25 (8.0%)	1.000
Ertapenem	0/29 (0.0%)	0/64 (0.0%)	-	0/33 (0.0%)	2/25 (8.0%)	0.181
Meropenem	0/29 (0.0%)	1/64 (1.6%)	1.000	0/33 (0.0%)	0/25 (0.0%)	-
Aztreonam	0/29 (0.0%)	0/64 (0.0%)	-	3/33 (9.1%)	2/25 (8.0%)	1.000
Amikacin	0/29 (0.0%)	0/62 (0.0%)	-	0/33 (0.0%)	0/25 (0.0%)	-
Gentamicin	0/29 (0.0%)	0/62 (0.0%)	-	0/33 (0.0%)	1/25 (4.0%)	0.431
Tobramycin	0/29 (0.0%)	1/62 (1.6%)	1.000	0/33 (0.0%)	1/25 (4.0%)	0.431
Ciprofloxacin	2/29 (6.9%)	3/64 (4.7%)	0.645	3/33 (9.1%)	4/25 (16.0%)	0.45
Levofloxacin	1/29 (3.4%)	2/64 (3.1%)	1.000	3/33 (9.1%)	3/25 (12.0%)	1.000
Trimethoprim–sulfamethoxazole	0/29 (0.0%)	1/64 (1.6%)	1.000	4/33 (12.1%)	3/25 (12.0%)	1.000
Tetracycline	8/29 (27.6%)	15/64 (23.4%)	0.668	32/33 (97.0%)	23/25 (92.0%)	0.572
Nitrofurantoin	7/29 (24.1%)	4/62 (6.5%)	0.033[4]	1/33 (3.0%)	0/25 (0.0%)	1.000

[1]Overange: *E. coli* density exceeds 1,479 MPN per 100 mL; [2]NS: Not susceptible to antibiotic; [3] p: Unadjusted p-values from Fisher Exact tests (if any cell count is ≤ 5) or Chi-Square tests and is not adjusted for multiple comparisons. [4] 0.033: Unadjusted p-value without Holm-Bonferroni correction, which adjusts p to 0.495.

Table S9. The sensitivity, specificity, and area under the ROC curve for characterizing the diagnostic performance of individual antibiotic non-susceptibility for predicting multidrug resistance (not susceptible ≥ 3 classes) using all studied isolates (*N* = 149), including non-*E. coli*, from both tetracycline-treated and untreated media.

**Class**	**Antibiotic**	**Sensitivity % (95% C.I.)**	**Specificity % (95% CI)**	**ROC Area**
Penicillins	Ampicillin	100.0 (90.3–100.0)	92.0 (85.4–96.3)	0.96
β-Lactam / β-Lactam Inhibit. Combination	Ampicillin–sulbactam	77.8 (60.8–89.9)	99.1 (95.2–100.0)	0.88
	Amoxicillin–clav. acid	55.6 (38.1–72.1)	100.0 (96.8–100.0)	0.78
	Piperacillin–tazobactam	NC	NC	NC
1st CEP	Cefazolin	80.6 (64.0–91.8)	99.1 (95.2–100.0)	0.9
2nd CEP	Cefotetan	36.1 (20.8–53.8)	100.0 (96.8–100.0)	0.68
3rd CEP	Ceftriaxone	22.2 (10.1–39.2)	100.0 (96.8–100.0)	0.61
	Ceftazidime	38.9 (23.1–56.5)	100.0 (96.8–100.0)	0.69
	Cefotaxime	13.9 (4.7–29.5)	100.0 (96.8–100.0)	0.57
4th CEP	Cefepime	13.9 (4.7–29.5)	100.0 (96.8–100.0)	0.57
Carbapenems	Ertapenem	2.8 (0.1–14.5)	99.1 (95.2–100.0)	0.51
	Meropenem	0.0 (0.0–9.7)	99.1 (95.2–100.0)	0.5
Monobactams	Aztreonam	13.9 (4.7–29.5)	100.0 (96.8–100.0)	0.57
Amino-glycosides	Amikacin	NC	NC	NC
	Gentamicin	2.8 (0.1–14.5)	100.0 (96.8–100.0)	0.51
	Tobramycin	5.6 (0.7–18.7)	100.0 (96.8–100.0)	0.53
Fluoroquinolones	Ciprofloxacin	25.0 (12.1–42.2)	97.3 (92.4–99.4)	0.61
	Levofloxacin	22.2 (10.1–39.2)	99.1 (95.2–100.0)	0.61
Folate Pathway Inhibitor	Trimethoprim–sulfamethoxazole	22.2 (10.1–39.2)	100.0 (96.8–100.0)	0.61
Tetracycline	Tetracycline	69.4 (51.9–83.7)	53.1 (43.5–62.5)	0.61
Nitrofurans	Nitrofurantoin	27.8 (14.2–45.2)	98.2 (93.8–99.8)	0.63

Table S10. A comparison in the prevalence of non-susceptibility among all isolates recovered, including non-*E. coli,* to various antibiotics (ABX), including non-*E. coli*, from both tetracycline-treated and untreated media.

ABX	Untreated (No TE) in Media						TE Treated Media	
	ColiGlow		Colilert		Crude	Adj.	ColiGlow + TE	
	N	NS (%)	*N*	NS (%)	p^1	p^2	*N*	NS (%)
AM	70	23 (32.9%)	23	3 (13.0%)	0.106	1.000	58	19 (32.8%)
A/S	70	15 (21.4%)	23	2 (8.7%)	0.224	1.000	58	12 (20.7%)
AUG	70	14 (20.0%)	23	1 (4.4%)	0.105	1.000	58	5 (8.6%)
P/T	70	0 (0.0%)	23	0 (0.0%)	--	--	58	0 (0.0%)
CFZ	70	17 (24.3%)	23	1 (4.4%)	0.037	0.518	58	12 (20.7%)
CTN	68	10 (14.7%)	23	0 (0.0%)	0.06	0.780	58	3 (5.2%)
CAX	70	1 (1.4%)	23	1 (4.4%)	0.435	1.000	58	6 (10.3%)
CAZ	70	5 (7.1%)	23	1 (4.4%)	1.000	1.000	58	8 (13.8%)
CFT	70	0 (0.0%)	23	0 (0.0%)	--	--	58	5 (8.6%)
CPE	70	0 (0.0%)	23	0 (0.0%)	--	--	58	5 (8.6%)
ETP	70	0 (0.0%)	23	0 (0.0%)	--	--	58	2 (3.4%)
MER	70	1 (1.4%)	23	0 (0.0%)	1.000	1.000	58	0 (0.0%)
AZT	70	0 (0.0%)	23	0 (0.0%)	--	--	58	5 (8.6%)
AK	68	0 (0.0%)	23	0 (0.0%)	--	--	58	0 (0.0%)
GM	68	0 (0.0%)	23	0 (0.0%)	--	--	58	1 (1.7%)
TO	68	1 (1.5%)	23	0 (0.0%)	1.000	1.000	58	1 (1.7%)
CP	70	5 (7.1%)	23	0 (0.0%)	0.328	1.000	58	7 (12.1%)
LVX	70	3 (4.3%)	23	0 (0.0%)	0.572	1.000	58	6 (10.3%)
T/S	70	1 (1.4%)	23	0 (0.0%)	1.000	1.000	58	7 (12.1%)
TE	70	22 (31.4%)	23	1 (4.4%)	0.011	0.165	58	55 (94.8%)
FD	68	10 (14.7%)	23	1 (4.4%)	0.279	1.000	58	1 (1.7%)
MDR	68	19 (27.9%)	23	1 (4.4%)	0.009	--	58	16 (27.6%)
XDR	68	0 (0.0%)	23	0 (0.0%)	--	--	58	1 (1.7%)

[1] *p:* Unadjusted *p*-values from Fisher Exact tests.
[2] *p:* Holm-Bonferroni adjusted *p*-values from Fisher Exact tests accounting for the number of antibiotics assessed for a difference in the prevalence of non-susceptibility between the two untreated media types.

Table S11. A comparison of *E. coli* species identification organized by the starting media from which the isolate was recovered.

TE Treatment	Media Type	N	*E. coli* n (%)	Not *E. coli* n (%)	P^1
TE Treated	Colilert + TE	58	53 (91%)	5 (9%)	--
No TE Treatment	Coliglow	70	56 (80%)	14 (20%)	*0.341*
	Colilert	23	21 (91%)	2 (9%)	
Overall	--	151	130 (86%)	21 (14%)	--

[1] *p: p*-value from Fisher Exact test.

Table S12. The number of recovered *E. coli* and non-*E. coli* isolates from each media type and their frequency of non-susceptibility to 13 antibiotic categories for determining multidrug resistance (MDR); whereby MDR is defined as non-susceptibility to at least one antibiotic in three or more antibiotic categories. *None were observed for nine categories.

Origin of Recovered Isolate	No.	No. of Antibiotic Categories with Non-Susceptibility										No. (%MDR)
		0	1	2	3	4	5	6	7	8	10	
ColiGlow	68	34	12	3	3	9	4	3	-	-	-	19 (27.94)
Colilert	23	18	4	-	-	1	-	-	-	-	-	1 (4.35)
ColiGlow + TE	58	-	37	5	3	5	3	-	1	3	1	16 (27.59)
Total	149	52	53	8	6	15	7	-	1	3	1	36 (24.16)

Table S13. The number of recovered *E. coli* isolates from each media type and their frequency of non-susceptibility to 13 antibiotic categories for determining multidrug resistance (MDR); whereby MDR is defined as non-susceptibility to at least one antibiotic in three or more antibiotic categories. *None were observed for nine categories.

Origin of Recovered Isolate	No.	No. of Antibiotic Categories with Non-Susceptibility										No. (%MDR)
		0	1	2	3	4	5	6	7	8	10	
ColiGlow	54	33	12	-	2	6	1	-	-	-	-	9 (16.67)
Colilert	21	18	2	-	-	1	-	-	-	-	-	1 (4.76)
ColiGlow + TE	53	-	35	5	3	5	-	-	1	3	1	13 (24.53)
Total	128	51	49	5	5	12	1	-	1	3	1	23 (17.97)
